# Hemorrhagic encephalopathies and myelopathies in dogs and cats: a focus on classification

**DOI:** 10.3389/fvets.2024.1460568

**Published:** 2024-10-28

**Authors:** Koen M. Santifort, Simon Platt

**Affiliations:** ^1^IVC Evidensia Small Animal Referral Hospital Arnhem, Neurology, Arnhem, Netherlands; ^2^IVC Evidensia Small Animal Referral Hospital Hart van Brabant, Neurology, Waalwijk, Netherlands; ^3^Vet Oracle Teleradiology, Norfolk, United Kingdom

**Keywords:** hemorrhagic infarct, hematomyelia, vascular malformation, T2* gradient echo, susceptibility-weighted imaging

## Abstract

The prevalence of hemorrhagic diseases of the central nervous system of dogs and cats is low compared to other diseases such as neoplasia and inflammation. However, the clinical consequences can be devastating. Several etiological and localization-based classification systems have been reported for intracerebral and spinal cord hemorrhage or hematomyelia in humans but similar systems do not exist in veterinary medicine. The authors propose an etiologic classification system for both intraparenchymal hemorrhagic encephalopathy and myelopathy following a review of the literature detailing the presentation, diagnosis, therapy, and prognosis of these diseases. A summary of the investigative and therapeutic approach to these cases is also provided.

## Introduction

Hemorrhagic encephalopathies and myelopathies in dogs and cats can have various etiologies and localizations. In human medicine, classifications based on etiology are commonly used and have been subject to change over time. In veterinary neurology, etiological classifications are not specifically agreed upon.

Classification can be important because it may contribute to a better understanding of underlying pathology, facilitate comparability between patient populations at different care facilities (e.g., for research purposes), and improve patient care by guiding diagnostic, therapeutic, and preventative measures.

Classification systems in human medical literature have been developed and employed, providing the opportunity to study both hemorrhagic encephalopathies and myelopathies retrospectively and prospectively with regard to associations with outcomes or effects of therapy.

While many pathological processes may involve some degree of hemorrhage, not all those encephalopathies or myelopathies with macro-or microscopic hemorrhage should be termed ‘hemorrhagic encephalopathies/myelopathies’ *per se*. For the purpose of this review, we define hemorrhagic encephalopathies and myelopathies as those encephalopathies and myelopathies where hemorrhage is an inherent part of the primary pathology, (suspected) main cause of the clinical signs, and/or dominant feature on diagnostic imaging or post-mortem examinations.

This review aims to 1/discuss classifications of hemorrhagic encephalopathies and myelopathies in humans, 2/summarize reported etiologies of canine and feline intraparenchymal hemorrhagic encephalopathies and myelopathies, 3/propose simple, easily applied classification systems based on etiology and localization, and 4/briefly review diagnostic, therapeutic, and prognostic considerations, based on veterinary and human literature.

## Hemorrhagic encephalopathies

### Etiological classification

#### Human

In human medical literature, several etiological classification systems have been reported for intracerebral hemorrhage specifically. The three most recently reported of these are chronologically included and discussed below.

- SMASH-U (1)

This classification system was devised to be simple and clinically practical. The six categories of disease are structural vascular lesions (S), medication-associated (M), amyloid angiopathy (A), systemic disease (S), hypertension (H), and undetermined (U). Traumatic and tumor-associated etiologies were excluded. The undetermined (U) category was not specified further. The authors proposing this system evaluated the reproducibility (interobserver agreement, kappa of 0.89) and prognostic value in a retrospective study on 1,013 patients with signs or symptoms of >24 h duration. Several categories had odds ratios significantly different from 1 (either <1 or > 1) for mortality at 3 months post-diagnostic procedures. Based on this, the authors concluded that the classification system as a whole has prognostic value. In the evaluation of this system, the authors did not account for the possibility of more than 1 category and strict definitions were mostly lacking.

- H-ATOMIC classification (2)

This classification system was formed based on the frequency of diagnoses in human medicine and includes 7 categories of disease which are hypertension (H), cerebral amyloid angiopathy (A), tumor (T), oral anticoagulants (O), vascular malformation (M), infrequent causes (I), and cryptogenic (C). This system also does not account for traumatic hemorrhagic encephalopathies, as this etiology was specifically excluded. The authors acknowledged that more than 1 category may apply to a single patient. The authors included three levels of certainty for disease categorization: definite (1), probable (2), and possible (3). For instance, this leads to a classification of T1 for confirmed tumor-associated hemorrhagic encephalopathy. The cryptogenic category was concluded based on the exclusion of the other categories. The ‘infrequent causes’ category was broad and based on the relatively low prevalence of these causes in humans with hemorrhagic encephalopathies. This category included but was not limited to disorders such as intracranial aneurysms (congenital, mycotic, other causes), venous thrombosis, illegal drug-or alcohol-associated, hypertensive crisis, and pituitary apoplexy. Along with this classification system, guidelines for diagnostic protocols and minimal work-up were published, including definitions of the specific requirements to be assigned to a category of this classification. Notably, the etiologic category of ‘oral anticoagulants (O)’ is not considered a real cause of hemorrhage in the brain, but rather a risk factor by other authors (3).

- CLAS-ICH (3)

CLAS-ICH stands for ‘classification system for intracerebral hemorrhage’ and is an imaging-based system. The system was devised by the authors based on recent neuroimaging advancements, recent data regarding the epidemiology (prevalence) of intracerebral hemorrhage, and advancements in cerebral small vessel disease (SVD) phenotyping. The categories were also defined keeping in mind potential clinical relevance, i.e., the system had to be simple and yield outcomes of possible relevance for acute patient management. Finally, categories were defined accounting for overlapping pathogenesis and prognosis that may affect treatment strategies.

Five categories are included: arteriolosclerosis, cerebral amyloid angiopathy, mixed small vessel disease (SVD), other rare forms of SVD (genetic SVD and others), and secondary causes (macrovascular causes, tumor, and other rare causes). Traumatic causes are not accounted for and were not included in the study for evaluation of this system. Every patient is scored in each category according to the level of diagnostic evidence: (1) well-defined ICH subtype; (2) possible underlying disease; and (0) no evidence of the disease Unlike the previous two systems, patients are classified for each of the categories. This is based on the level of certainty or ‘diagnostic evidence’ and graded as well-defined; (1) possible underlying disease; (2) and no evidence of disease (0). This system therefore acknowledges that multiple etiologies may be relevant in a single patient. This system was studied in two cohorts of 113 and 203 patients, respectively. Interobserver agreement was very good to perfect (kappa of 0.86–1.00).

#### Veterinary literature and classification proposal

According to the veterinary neurology acronym of VITAMIN D, hemorrhagic encephalopathy has been reported for each of the abbreviated categories (Table 1). In the literature documenting these disorders, hemorrhage is often mentioned as an aside. That is, hemorrhage was deemed to be a secondary feature of the primary disorder. The hemorrhage itself may not be responsible for clinical signs or a specific target of treatment. On the other hand, hemorrhage as a secondary feature of another primary disorder may be a pivotal complication with possibly fatal consequences.

**Table 1:** Hemorrhagic encephalopathies classified into VITAMIN D categories, including example references.

Vitamin D category	Examples	References
Vascular	Hemorrhagic stroke Hemorrhagic transformation of ischemic stroke Cerebral amyloid angiopathy / beta-amyloid angiopathy	(6, 8, 13, 122–125)
Idiopathic		(7, 12, 33)
Inflammatory-infectious	Viral infections (e.g., canine adenovirus) Feline infectious peritonitis Protozoal encephalitis Bacterial encephalitis	(126)
Inflammatory-immunemediated	Meningoencephalitis of unknown origin	(127–129)
Traumatic	Type of trauma (e.g., traffic accident, bite trauma) With/without cranial vault fractures With/without pre-existing brain disorder	(24, 27)
Toxic/Drug-associated	Lead intoxication Rodenticide poisoning	(130–134)
Anomalous	Vascular malformations	(13)
Metabolic	Various endocrinological disorders (associated with hemorrhagic diathesis or hypertension) Electrolyte imbalance (e.g., hypernatremia)	(32, 135)
Neoplastic	Lymphoma Hemangiosarcoma Glioma	(100, 136–141)
Nutritional	Thiamine deficiency	(142)
Degenerative	Fibrinoid leukodystrophy	(143, 144)
Other	Hemorrhagic diathesis Heat stroke Hypertension-associated	(32, 33, 70, 145–150)

As gleaned from the human etiological classification system, traumatic causes are often excluded or seen as an entirely separate and clearly identifiable (from the history or presentation) cause. However, in veterinary medicine, unobserved trauma and a lack of trauma in the history due to a lack of the ability to self-report is much more common. We therefore include trauma as an etiological category in our classification proposal.

For the proposed veterinary classification system, we elected to include the considerations above. As the aim was for the classification system to be simple and easily applied, three broad categories were defined, with sub-categories listed according to the well-known VITAMIN D scheme that veterinary neurologists, neuroradiologists, and neurosurgeons will be aware of (Table 1).

The classification system (Table 2) includes the following categories:

- Primary hemorrhagic encephalopathy

**Table 2:** Veterinary classification scheme of hemorrhagic encephalopathies as suggested by the authors.

Category	Examples
Primary	Idiopathic hemorrhagic stroke Idiopathic hemorrhagic encephalopathy Cerebral amyloid angiopathy/beta-amyloid angiopathy Hypertension associated hemorrhagic encephalopathy
Secondary	Vascular malformations Neoplasia (primary brain tumors or metastatic disease) Vasculitis associated or not with meningoencephalitis (infectious or immune-mediated) Coagulopathies (hemorrhagic diathesis of any cause) Infarcts with hemorrhagic transformation Metabolic/nutritional/degenerative/toxic disorders Heat stroke
Traumatic	Specify type of trauma (e.g. traffic accident, bite trauma) Subcategories: With/without cranial vault fractures With/without pre-existing brain disorder (e.g., hydrocephalus)

**Figure 1:**
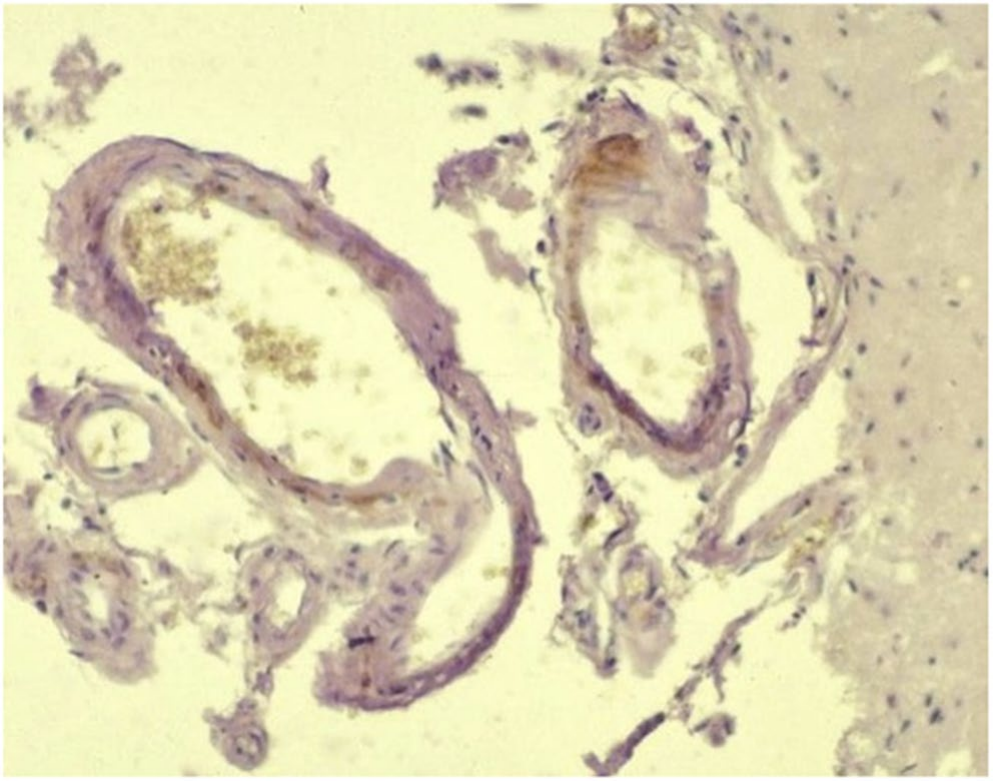
Beta-amyloid angiopathy: amyloid deposition in the wall of a leptomeningeal vessel (Avidin–biotin peroxidase complex (ABC) method for beta-amyloid protein, Mayer’s hematoxylin counterstain) – courtesy of M. Pumarola, Unit of Compared and Murine Pathology, Department of Animal Medicine and Surgery, Faculty of Veterinary Medicine, Universitat Autònoma de Barcelona, Barcelona, Spain.

**Figure 2:**
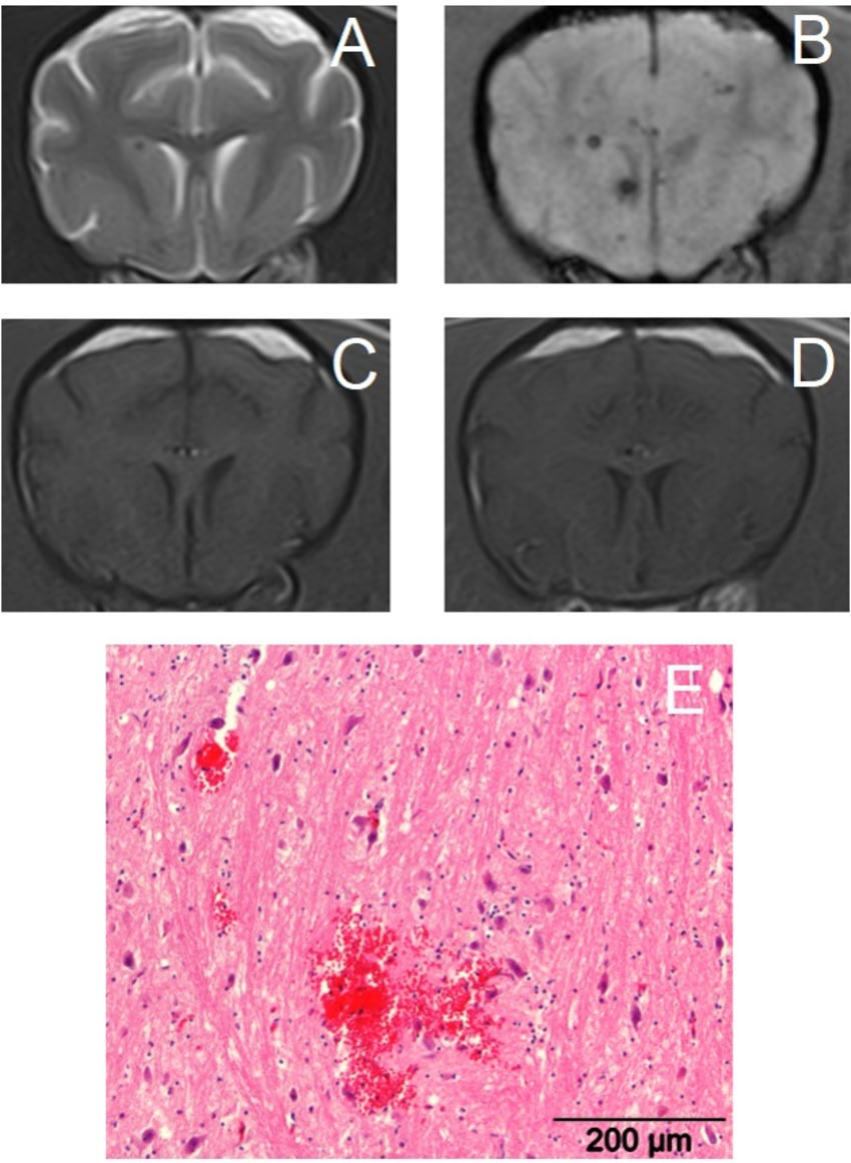
Transverse magnetic resonance images of the brain of a 14-year-old crossbreed dog with a primary hemorrhagic encephalopathy – hypertension-associated cerebral microbleeds. All images are at the level of the head of the caudate nuclei. Left is on the right of the images. (A): T2-weighted, (B): Susceptibility-weighted, (C): T1-weighted, (D): T1-weighted post-contrast, (E): photograph of hematoxylin and eosin stained brain tissue with an acute cerebral microbleed – courtesy of W. Bergman, Veterinary Pathology Diagnostic Center, Department of Biomedical Health Sciences, Faculty of Veterinary Medicine, Utrecht University, Utrecht, The Netherlands.

**Figure 3:**
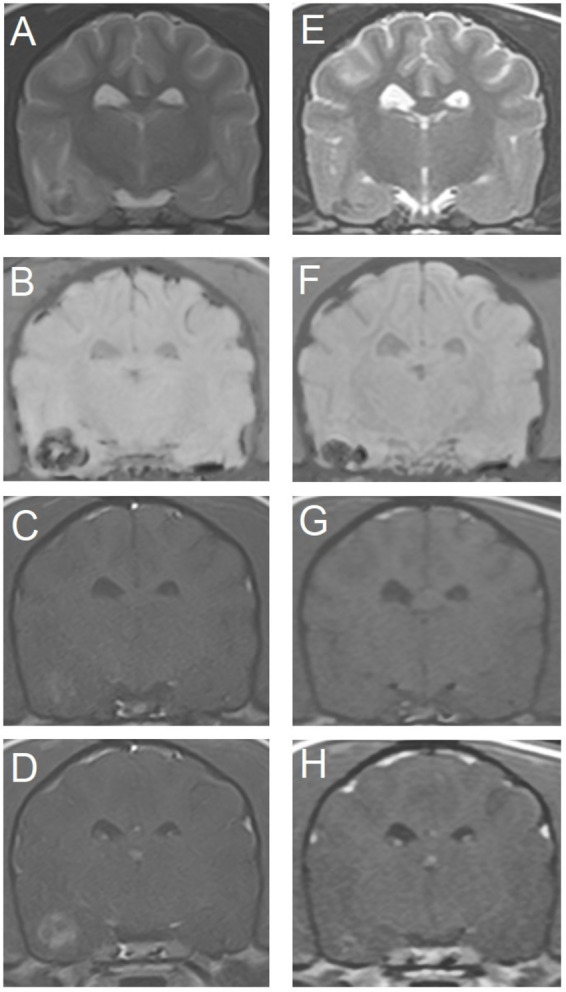
Transverse magnetic resonance images of the brain of an 11-year-old miniature Dachshund with a primary hemorrhagic encephalopathy – idiopathic hemorrhagic stroke. All images are at the level of the temporal lobes and caudal aspect of the interthalamic adhesion. Left is on the right of the images. A–D are images from a study 3 days after peracute onset of clinical signs. E–H are images from a follow-up study performed 4 weeks later. (A,E) T2-weighted. (B,F) Susceptibility-weighted. (C,G) T1-weighted. (D,H) T1-weighted post-contrast.

Note: ‘cerebrovascular accident (CVA)’ is a term that is used in literature interchangeably with ‘stroke’ and may refer to ischemic stroke or hemorrhagic stroke.

This category includes vascular disorders (e.g., cerebral amyloid angiopathy/beta-amyloid angiopathy) (4–6) (Figure 1), excluding vasculitis and malformations (included in the secondary category), hypertension-associated hemorrhage (Figure 2), idiopathic hemorrhagic stroke (i.e., no underlying causes identified) (Figure 3), and idiopathic hemorrhagic encephalopathy. Cerebral microbleeds are included in this category and may be associated with hypertension or angiopathy (microvascular disease). In dogs, they has been associated with proteinuria (7–11). Hemorrhagic stroke would be characterized by acute to peracute onset of clinical signs with localization to any part of the brain and diagnostic or histopathological findings of hemorrhage (12). Idiopathic hemorrhage would include those cases in which hemorrhage is found, but the clinical signs are not reflective of a stroke (i.e., there is no acute to peracute history) and further tests including diagnostic imaging and histopathology did not reveal any specific causes.

- Secondary hemorrhagic encephalopathy

**Figure 4:**
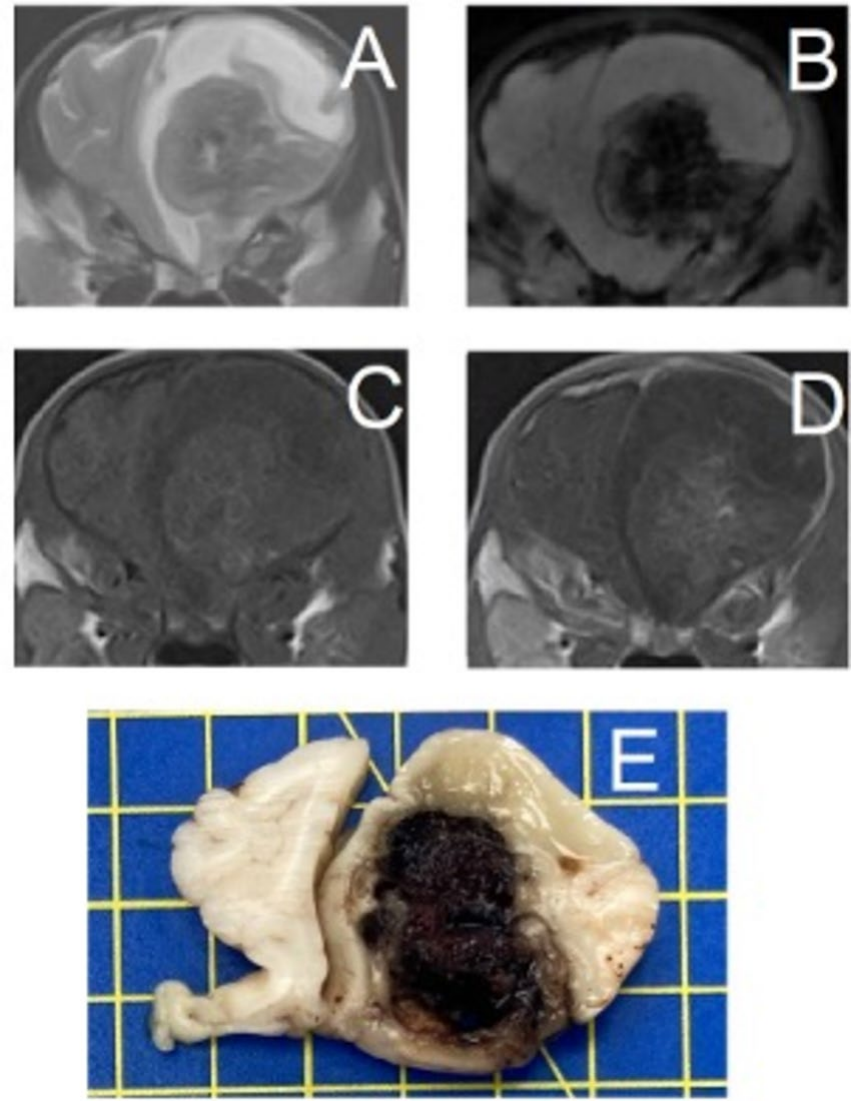
Transverse magnetic resonance images and formalin-fixed transverse slab of the brain of a 2-month-old Spanish Waterdog with a secondary hemorrhagic encephalopathy – secondary to a ruptured cavernous hemangioma. All images are at the level of the frontal lobes just rostral to the septum telencephali. Left is on the right of the images. (A) T2-weighted, (B) Susceptibility-weighted, (C) T1-weighted, (D) T1-weighted post-contrast, (E) photograph of formalin-fixed slab of brain tissue (yellow squares are 1×1 cm) – courtesy of N. Ankringa, Veterinary Pathology Diagnostic Center, Department of Biomedical Health Sciences, Faculty of Veterinary Medicine, Utrecht University, Utrecht, The Netherlands.

**Figure 5:**
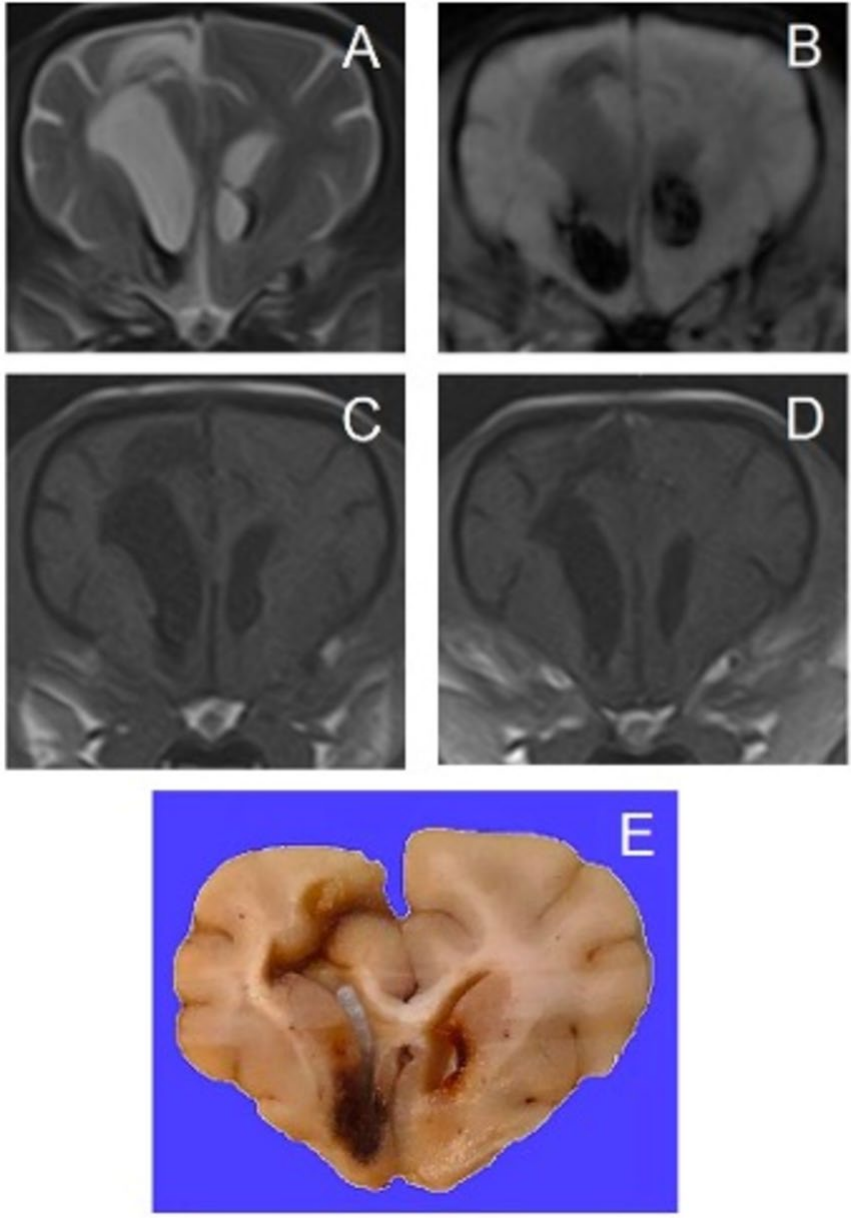
Transverse magnetic resonance images and formalin-fixed transverse slab of the brain of a 7-year-old Pomeranian with a secondary hemorrhagic encephalopathy – secondary to multifocal, non-symmetrical, lympho-plasma-histiocytic, necrotizing encephalitis of unknown origin. All images are at the level of the septum telencephali. Left is on the right of the images. (A) T2-weighted, (B) Susceptibility-weighted, (C) T1-weighted, (D) T1-weighted post-contrast, (E) photograph of formalin-fixed slab of brain tissue – courtesy of G.C.M. Grinwis, Veterinary Pathology Diagnostic Center, Department of Biomedical Health Sciences, Faculty of Veterinary Medicine, Utrecht University, Utrecht, The Netherlands.

This category includes vascular malformations (13) (Figure 4), neoplasia (primary brain tumors or metastatic disease), vasculitis, meningoencephalitis (infectious or immune-mediated) (Figure 5), coagulopathies (hemorrhagic diathesis of any cause), and infarcts with hemorrhagic transformation (i.e., ischemic stroke with hemorrhagic transformation). Metabolic, nutritional (e.g., thiamin deficiency), degenerative, or toxic (e.g., lead intoxication) disorders and heat stroke may also feature hemorrhagic lesions in the brain.

- Traumatic hemorrhagic encephalopathy

**Figure 6:**
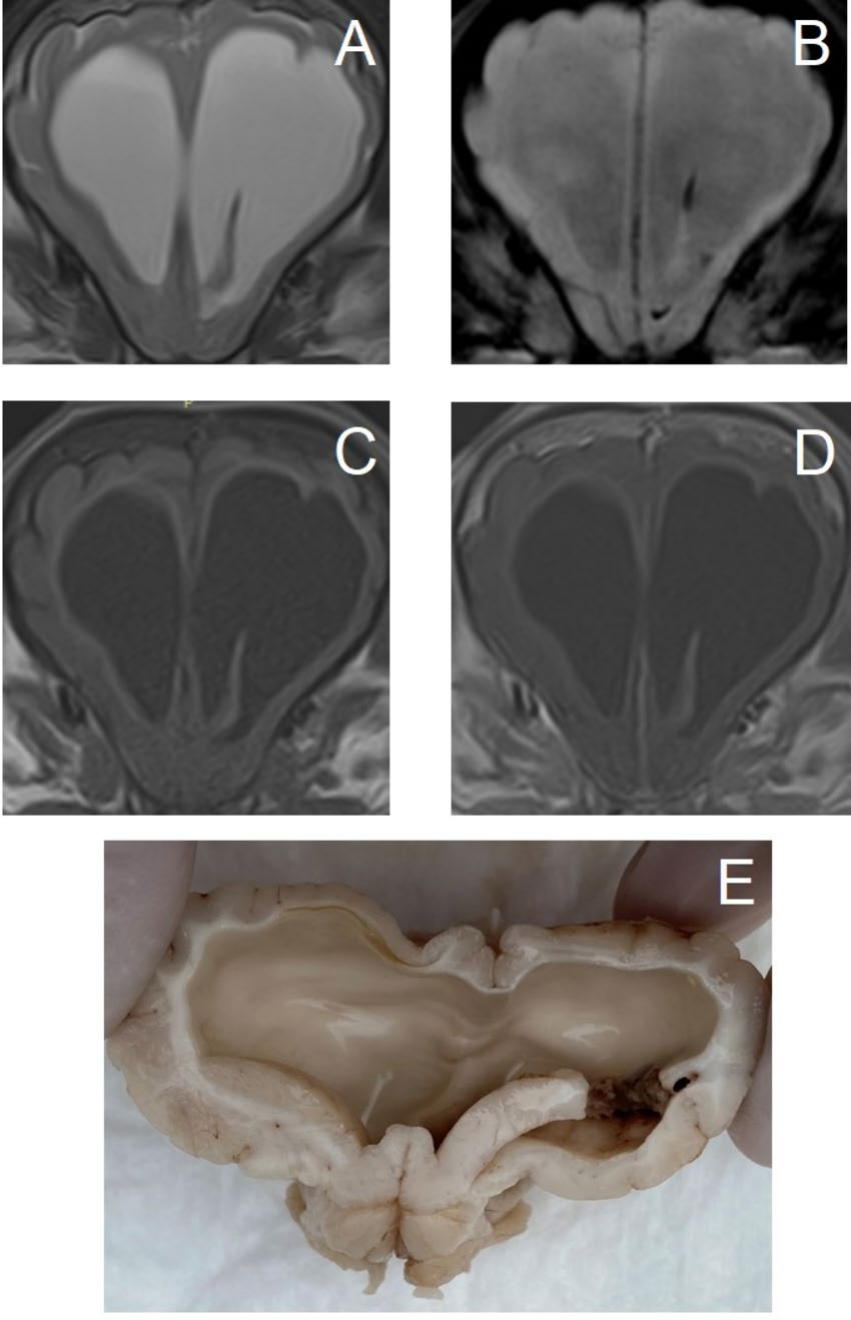
Transverse magnetic resonance images and formalin-fixed transverse sectioned brain of a 4-month-old Boxer with a traumatic hemorrhagic encephalopathy – trauma to the head (minor blunt trauma: bumped its head against a wall) without cranial vault fractures, with pre-existing obstructive hydrocephalus due to congenital mesencephalic aqueduct stenosis. (A–D) images are at the level of the septum telencephalic. Left is on the right of the images. (A) T2-weighted, (B) Susceptibility-weighted, (C) T1-weighted, (D) T1-weighted post-contrast, (E) caudorostral mirrored photograph of formalin-fixed sectioned brain at the level of the head of the caudate nuclei (the hemorrhage is on the left side, mirror-image for comparison with MRI).

This is separately classified, as mechanical injury to brain tissue and blood vessels results in hemorrhage as a complicating factor. The type of trauma should be specified if possible (e.g., traffic accident, bite trauma). Subcategories include those with or without cranial vault fractures and those with or without pre-existing brain disorders (e.g., hydrocephalus (Figure 6), or brain neoplasia).

Selected examples of some of these categories are included in Figures 2–6.

### Localization

Intracranial hemorrhage may be classified based on localization. For completeness, we include all possible intracranial localizations of hemorrhage below.

However, this review focuses on intraparenchymal hemorrhage, the most common localization in small animal clinical neurology. This ‘classification system’ is not species-specific (i.e., applicable to humans, dogs, cats, *et cetera*).

Intracranial hemorrhage may be located in any of the following locations (12, 14–21):

- Extraparenchymal hemorrhage
  - Epidural hemorrhage: outside of the dura mater
  - Subdural hemorrhage: under the dura mater, outside of the arachnoid membrane
  - Subarachnoid hemorrhage: within the subarachnoid space
  - Subpial hemorrhage: under the pia mater
  - Subependymal hemorrhage: under the ependymal lining of the ventricles
  - Intraventricular hemorrhage: within the ventricular system
- Intraparenchymal hemorrhage: within the brain parenchyma

One, two, or multiple of these localization may be involved at once. This list of localizations can be elaborated even more extensively and details can be added to the localizations mentioned. For instance, for intraparenchymal hemorrhage, one can localize it to specific structures or regions and/or gray versus white matter; and for intraventricular hemorrhage, the specific parts of the ventricular system that are involved should be mentioned.

The value of a localization-based classification lies mainly in the option of surgically addressing the hemorrhagic component itself. For instance, an extraparenchymal hemorrhage or epidural hemorrhage may be cleared via craniotomy, flushing, and suction.

Some of the localizations have not been specifically covered in previous reviews on hemorrhagic brain disorders in small animals but can be found in human literature, particularly in neonates [e.g., subependymal hemorrhage (17)]. Whether or not some of the above localizations have or have not been specifically reported is not particularly relevant as long as readers are aware of the theoretical localizations of the hemorrhagic components. Intraparenchymal hemorrhages are most commonly reported in dogs and cats. Extra-parenchymal hemorrhage in small animals is most commonly the result of trauma (20–22).

### Clinical signs

Clinical signs related to hemorrhagic encephalopathies are primarily dependent on the location of the hemorrhage within the brain tissue, the extent of hemorrhage, associated pathology, secondary effects (e.g., mass effect), and time frame (e.g., acute versus chronic phase). Therefore, signs vary greatly and may include abnormal mentation, abnormal behavior, epileptic seizures, cranial nerve deficits, abnormal postures, paresis, proprioceptive deficits, ataxia, hyperesthesia, and abnormal sensation.

### Diagnostic considerations

In general, the history will be instrumental in excluding the traumatic category of hemorrhagic encephalopathy. If there is no owner or no history of trauma, trauma cannot be excluded immediately. Therefore if in doubt, this possibility should be taken into consideration for both diagnostic decision-making and treatment.

For most cases of hemorrhagic encephalopathies, diagnostic imaging will be necessary for *in vivo* diagnosis. Magnetic resonance imaging (MRI) would be the modality of choice in the majority of cases, excluding acute traumatic cases, in which computed tomography (CT) offers numerous advantages, including shorter scan time, no need for anesthesia (i.e., sedation or even non-sedated studies can be performed if patients are immobile), and better sensitivity for cranial vault fractures (23). Nevertheless, MRI has been shown to have prognostic value after traumatic brain injury in dogs (24).

In the human medical literature, the authors of the H-ATOMIC classification specifically detail diagnostic criteria and recommendations (2). These can also be considered for veterinary patients, to accurately classify hemorrhagic encephalopathies as well as to determine the best course of action for that patient. These broadly include (2, 25, 26):

- Neuroimaging (see above, CT versus MRI; consider angiography, especially for vascular malformations; consider follow-up imaging studies). For MRI studies, include T2*-weighted or susceptibility-weighted-imaging sequences (Figures 2–6). Follow-up imaging studies can be crucial to exclude or confirm suspicions of underlying disorders, such as neoplasia (Figure 3). The reader is referred to Arnold et al. for specific considerations for MRI studies (11).
- Blood tests (e.g., complete blood counts, differentiation, biochemistry (electrolytes, glucose, urea, creatinine), coagulation profiles (thromboelastography and D-dimers), endocrine tests)
- Cardiac ultrasound and electrocardiogram
- Thoracic and abdominal imaging studies (radiographs, ultrasound, CT – e.g. staging or looking for primary neoplastic disorders)
- Cerebrospinal fluid analysis (when no contraindications are present, preferably after diagnostic imaging and when hemostatic disorders are excluded)
- *In vivo* biopsy and histopathology and/or post-mortem examination. It must be noted that histopathological confirmation of a diagnosis is almost always required to obtain certainty about the diagnosis and thus the classification of hemorrhagic encephalopathies.

### Treatment considerations

As mentioned for diagnostic considerations, the possibility of trauma should be taken into consideration for treatment decisions. In cases of traumatic brain injury and hemorrhage, a peracute or acute and progressive onset of signs is expected and rapid treatment to preserve adequate cerebral perfusion and oxygenation is vital. Briefly, oxygen supplementation and fluid therapy are the pillars of achieving these goals. Aside from maintaining intravascular volume and blood pressure, decreasing intracranial pressure by means of infusion therapy (e.g., mannitol, hypertonic saline) or surgery (e.g., emergency craniotomy) are major considerations in the acute phase (22). Treatment for traumatic brain injury is reviewed extensively elsewhere and there is no current evidence for specific hemorrhage-focussed therapy in veterinary medicine (27).

For the treatment of primary hemorrhagic encephalopathy, aside from that associated with hypertension, no underlying causes can be specifically addressed and treatment focuses on the same principles as treatment for traumatic brain injury: preserve adequate cerebral perfusion and oxygenation. Any other complicating factors, such as epileptic seizures or metabolic derangements, should be adequately and swiftly treated. The main focus is thus to ‘buy time’ for the intracranial environment to achieve homeostasis and for the brain (as an organ) to slowly repair the damage and degrade the hematoma (s).

In those hemorrhagic encephalopathies where intracranial pressure is elevated acutely, and medical treatment does not result in adequate stabilization, consideration should be given to the surgical approach for potential evacuation of a hematoma. The goal remains the same, which is to achieve adequate cerebral perfusion and oxygenation, and thus prevent further damage and provide the opportunity of restoring brain function. For non-traumatic hemorrhagic (intraparenchymal) hemorrhage, there is currently no evidence-based indication for surgical treatment in veterinary medicine. Indication for surgical treatment should thus be based on best clinical judgment by the primary clinicians.

The treatment of secondary hemorrhagic encephalopathy also includes the above but will be supplemented with treatment for the specific underlying cause, if such treatment is possible. For instance, bacterial encephalitis will require treatment with antibiotics, and rodenticide poisoning (depending on the type) can be treated with vitamin K. In short, when underlying causes are identified, the treatment is aimed at addressing this underlying cause. For many of the possible etiologies, however, this may not be possible short-term (e.g., neoplasia) or not possible at all (degenerative disorders).

While treatment options for clinical veterinary patients are fairly limited, treatment considerations for human counterparts are reviewed and discussed extensively in the literature (25, 28, 29). Many of the specific treatment considerations for humans are not available or feasible (due to timing) for veterinary patients, while those that are lack firm evidence to support their routine implementation. Human hospitals frequently have dedicated stroke care units – a fact that underlines the importance of timing and protocols in the management of hemorrhagic stroke. Medical treatments include those focused on lowering blood pressure in cases of hypertension to stop active bleeding or reduce the odds of further bleeding, use of hemostatic agents, and anticoagulant reversal agents. Surgical treatment of intracranial hemorrhage in people is supported by evidence while some aspects do remain controversial, such as the exact timing of the surgery. Nonetheless, surgery within a matter of hours (<8 h) has been associated with improved outcomes (25, 28, 29).

### Prognostic considerations

In every peracute or acute presentation related to hemorrhagic encephalopathies (whether known immediately or evidenced by further tests later on), the modified Glasgow Coma Scale (MGCS) can be considered to be useful, at least in dogs (30, 31). The ability to assess numerically for deterioration or improvement longitudinally provides the clinician with a valuable tool in decision-making and owner communication. However, it must be kept in mind that, in essence, any such tool relies heavily on the most basic neurological clinical tests, and therefore repeated general physical and neurological examinations are the foundation of assessments of the effectiveness of initiated treatment and expected development.

There are no specific guidelines for prognostication of hemorrhagic encephalopathies in clinical veterinary patients in general, as there are many possible etiologies. For primary hemorrhagic encephalopathies, medium-and long-term prognosis may be very good when the animal survives the short-term as most hemorrhages will abate and resultant hematomas will be cleared eventually. Long-term sequelae (e.g., epilepsy) might negatively influence long-term outcomes or survival (e.g., if owners opt for euthanasia due to such complications), but has not been studied. If primary hemorrhagic encephalopathy is due to hypertension, the ability to treat for hypertension and any underlying disorders itself will influence the prognosis. For secondary hemorrhagic encephalopathies, prognosis will highly depend on the underlying cause. The presence of hemorrhage associated with neoplastic disorders has a very different prognosis from hemorrhage secondary to hemorrhagic diathesis related to *Angiostrongylus vasorum* infections, for instance (being generally worse for the former and good for the latter with short-term survival and appropriate treatment) (32–34).

From literature documenting numerous methods that can be of use to prognosticate patients with head trauma (24, 30, 31, 35–37), it is clear that even the most severely affected patients can show significant short-and long-term improvement. It is prudent to be cautious at early stages of presentation of any patient presented for traumatic hemorrhagic encephalopathy based on these observations. Specific prognostic factors and schemes that have been reported in the veterinary literature include MRI and CT grading schemes (e.g., presence of fractures, mass effect, herniation, compartmental involvement), Modified Glasgow Coma Scale and other clinical grading schemes, and point-of-care tests, such as blood glucose levels in dogs (hyperglycemia negatively associated with survival). However, these pertain to head trauma, not specifically those with or without hemorrhage, and not at all to non-traumatic hemorrhagic encephalopathies.

For humans, specific factors associated with outcome have been reported in the literature. These include the presence or absence of hypertension, localization and extension of hemorrhage, age, pathogenesis (i.e., underlying causes), functional and imaging-based scoring schemes, timing of treatment including surgery, *et cetera* (25, 28, 29, 38–41). Future studies in clinical veterinary patients may yield more useful prognostic indicators for clinicians to consider.

## Hemorrhagic myelopathies

### Etiological classification

#### Human

Intraspinal hemorrhage is a rare clinical entity in humans. In Jellinger’s classification, human hematomyelia (intramedullary hemorrhage) is divided into three etiological groups: traumatic, secondary, and idiopathic (42).

Trauma is usually associated with vertebral fractures and or luxations which are often concurrently easily visible on imaging studies. However, hematomyelia can be associated with spinal cord injury (SCI) without radiographic abnormalities (SCIWORA) (43). SCIWORA is a term that denotes objective clinical signs of posttraumatic spinal cord injury without evidence of fracture or malalignment on plain radiographs and computed tomography (CT) of the spine. SCIWORA is most commonly seen in children with a predilection for the cervical spinal cord due to the increased mobility of the cervical spine, the inherent ligamentous laxity, and the large head-to-body ratio during childhood (43). Since this is a fairly rare clinical entity, much of its exact pathophysiology remains unknown but it has been suggested that the shearing and stretching of the spinal cord caused by its motion relative to the bony vertebrae at impact might lead to the rupture of intraparenchymal vessels (44).

Up to one-third of non-traumatic cases are idiopathic and these cases are often termed spontaneous hematomyelia (45).

Secondary hematomyelia is mostly associated with spinal cord-specific disorders. Most commonly this has been associated with anticoagulant treatment such as warfarin or heparin (45, 46), but may also be the result of tumors, radiation therapy, or vascular malformations (47, 48). A rare cause of bleeding within the spinal cord is Gowers’ intra-syringal hemorrhage, which occurs from a sudden increase of pressure within the syringomyelia cavity and secondary vessel rupture (46).

#### Veterinary literature and classification proposal

Based on the veterinary literature, spinal cord hemorrhage can be initially divided into traumatic and non-traumatic causes (Table 3).

- Traumatic spinal hemorrhage (TSH) is suggested to be more common than non-traumatic causes and can include exogenous trauma such as vehicle-related accidents, falls and kicks, endogenous trauma which is related to disk extrusions, and iatrogenic trauma, e.g., after lumbar cerebrospinal fluid [CSF] acquisition.
  - Exogenous trauma

**Table 3:** Veterinary classification scheme for hemorrhagic myelopathies as suggested by the authors.

Category	Subcategory	Examples
	Traumatic spinal hemorrhage	Exogenous trauma	Automobile related, Falls, Kicks etc.
		Endogenous trauma	Disk Extrusion – compressive, non-compressive and intradural/intramedullary
			Atlanto-axial subluxation
Iatrogenic		
Non-traumatic spinal hemorrhage	Idiopathic	Primary hematomyelia
	Primary hemostatic disorders	Hemophilia
		Immune-mediated thrombocytopenia
	Secondary hemostatic disorders	*Angiostrongylus vasorum*
		Rodenticide poisoning
		Snake envenomation
	Vasculopathy	Vascular malformations
		Vasculitis Primary e.g.,Steroid-responsive meningitis-arteritis (SRMA) Secondary, e.g., Infectious meningomyelitis - *Leishmania*
		Hemorrhagic transformation of an ischemic lesion (e.g., fibrocartilaginous myelopathy)
		Radiation therapy
	Neoplasia	Hemangioblastoma, astrocytoma, ependymoma and metastatic hemangiosarcoma

**Figure 7:**
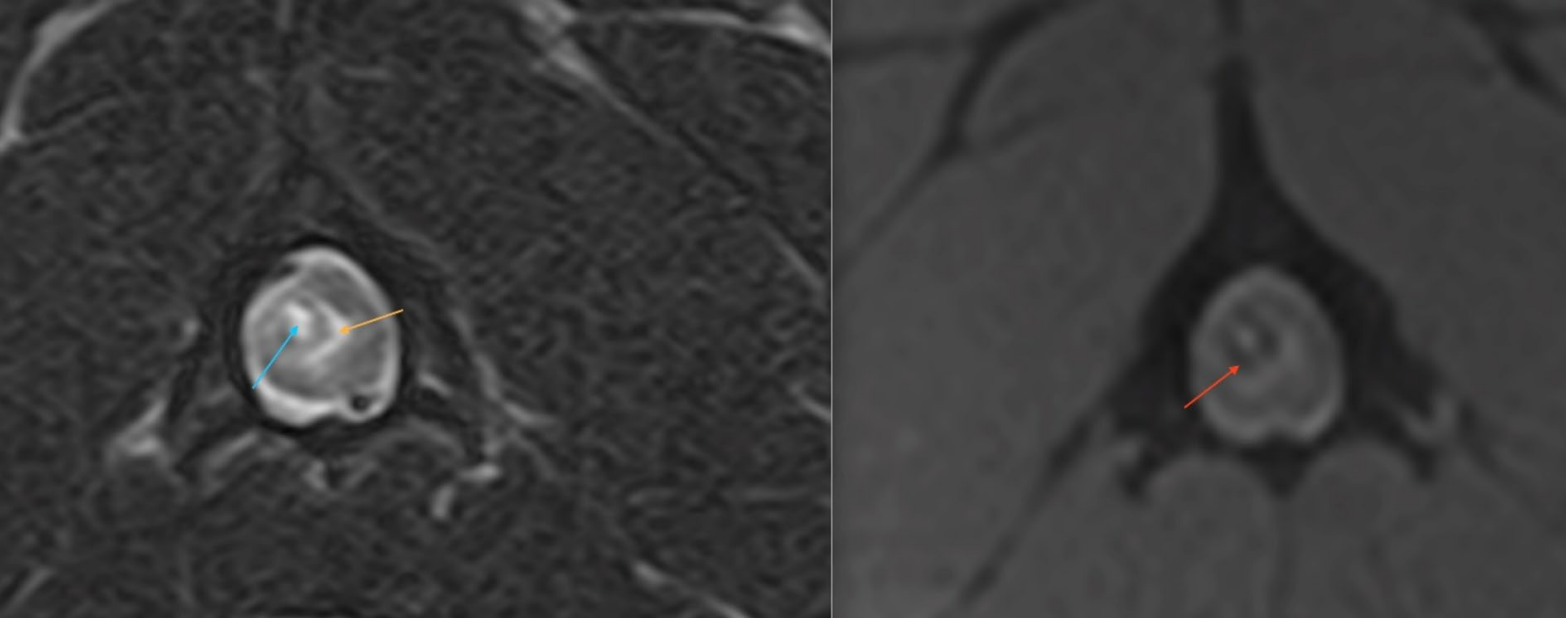
A transverse T2W (right side) and T2* (left side) MR image at the level of mid-body C2 of a 2-year-old French bulldog that had been hit by a car and presented with acute onset non-ambulatory tetraparesis. There were no evident osseous lesions noted on cervical vertebral radiographs. There is a relatively well-defined, ovoid intramedullary lesion within the right dorsal quadrant of the spinal cord which has a T2W and T2* hyperintense core (blue arrow), a hypointense rim (red arrow) and peripheral hyperintensity compatible with perilesional edema (orange). The lesion is compatible with a peracute traumatic intramedullary hematoma (49).

As for humans, this type of trauma is usually associated with vertebral fractures and or luxations easily visible on imaging studies. SCIWORA has been reported in veterinary medicine with two dogs and a cat documented to experience hematomyelia secondary to a road traffic accident and falls from a height; in all cases, radiography and MRI revealed no fracture or subluxation (44, 49) (Figure 7). Additionally, cervical hematomyelia has been reported in a cat secondary to a suspected bite injury to the neck, without imaging evidence of a fracture or luxation (50).

- Endogenous trauma
  - Intervertebral disk extrusion (IVDE)

**Figure 8:**
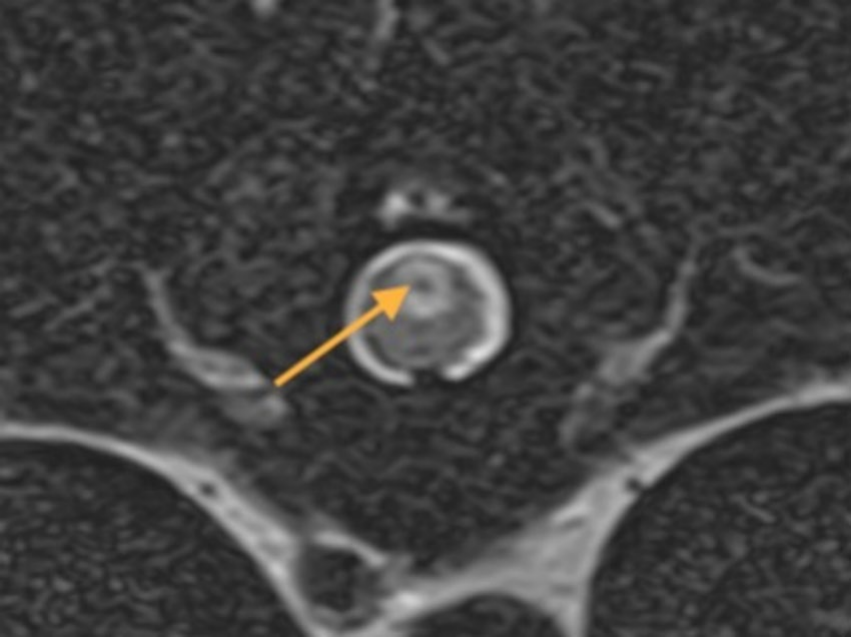
A transverse T2W MR image at T10-T11 of a 4-year-old Staffordshire Bull terrier with acute onset a traumatic non-compressive disk extrusion at T12-T13 and paraplegia. There is a well-defined ovoid dorsal midline intramedullary lesion with a T2W hypointense core (arrow) suggestive of hemorrhagic-necrotic material associated with the central canal. The MRI study was compatible with ascending hemorrhagic myelomalacia.

Although hemorrhage is a consequence of acute disk extrusion, whether it be compressive, non-compressive nucleus pulposus extrusion or intradural/intramedullary disk extrusion, it may also be a contributing factor to the further progression of traumatic myelomalacia, a significant structural disruption of the spinal cord secondary to intervertebral disk extrusion (51–54). Experimental studies suggest that the mechanism of this contribution involves detrimental biochemical effects of blood products on spinal cord tissue (55). The extent of intramedullary hemorrhage is significantly associated with the severity of spinal cord destruction at the site of the disk extrusion (56). In severe canine SCI following IVDE, blood can enter the central canal, presumably after its mechanical disruption at the epicenter of the lesion, which frequently leads to massive distention of the central canal in adjacent and remote segments (Figure 8). This subsequently leads to the rupture of the central canal with extrusion of the hemorrhagic debris into the dorsal column area suggesting the presence of a driving force propelling this material rather than the spread of the hemorrhagic debris by passive leakage.

- Developmental anomalies

Similar to IVDE, the most common cervical vertebral developmental anomaly, atlanto-axial subluxation, can be responsible for traumatic damage to the overlying cord and in some cases is associated with hematomyelia, which can complicate prognosis (57).

- Iatrogenic

**Figure 9:**
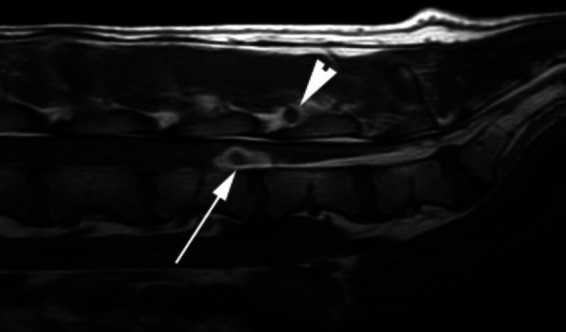
A sagittal T1W image of the caudal lumbar vertebral column of a 2 year-old Hungarian Vizsla 4 days after lumbar CSF acquisition and the subsequent onset of a flaccid paraparesis and tail paresis. There is a well-defined ovoid ventral intramedullary lesion centered over the L5-L6 intervertebral disk space (arrow). The lesion has a hypointense core and an irregular hyperintense periphery. A similar lesion is seen in soft tissues dorsal to the interarcuate space at this site (arrowhead).

Accidental or unintentional intraspinal hemorrhage unassociated with surgery has been reported with myelographic procedures, cerebrospinal fluid acquisition in addition to inadvertent thoracic intraspinal injection (58, 59). The origin of the hemorrhage in both circumstances is purported to be the puncture of a parenchymal vessel which results in a sudden onset of clinical signs within hours of the lumbar puncture (Figure 9). However, although computed tomographic examination of the spinal cord of dogs after lumbar myelography has confirmed the presence of intramedullary hemorrhage, it is not always associated with clinical signs (60).

- Non-traumatic spinal hemorrhage (NTSH) can be idiopathic or secondary to an underlying disease process or medical condition (61). Several medical conditions have been associated with NTSH in dogs, many of which are associated with an effect on the coagulation capabilities of the animal.
  - Primary hemostatic disorders have been associated with:
    - Hemophilia

Hemophilia is the most common congenital coagulation disorder affecting secondary hemostasis in humans (62). Hemophilia A is an X-linked deficiency in factor VIII and hemophilia B is caused by a deficiency in factor IX (63). Hematomyelia and hematorrhachis have been reported in juvenile dogs with hemophilia A and a cat with hemophilia B (64–67). CNS bleeds in humans with hemophilia has a prevalence of approximately 7.5% with no history of trauma in 67% of these cases; only 1% of these hemorrhages were intraspinal (68).

- Immune-mediated thrombocytopenia

A 4-year-old neutered female Bearded Collie has been reported with progressive tetraparesis, immune-mediated thrombocytopenia and presumed secondary spinal cord hemorrhage (69).

- Secondary hemostatic disorders have been reported in association with:
  - *Angiostrongylus vasorum* (*A. vasorum*)

Common in Europe but rare in the United States, *A. vasorum* is a metastrongylid nematode worm that infects dogs after ingesting snails or slugs. This often results in a secondary coagulopathy and pulmonary hemorrhage, but central nervous system hemorrhage has been reported (70). Thrombocytopenia, anemia and eosinophilia are frequently observed in infected dogs, and diagnosis is usually based on finding the larvae in feces (71). Anthelmintic therapy and supportive care are recommended but anaphylactic reactions have been suspected, triggered by the rapid release of a large amount of worm antigen into the blood as the worms were killed (70).

- Rodenticide toxicity

Warfarin competitively inhibits the vitamin K epoxide reductase complex 1, which is essential for activating vitamin K. Vitamin K is an essential cofactor for the hepatic synthesis of multiple clotting factors including II, VII, IX, and X and factors protein C and protein S. As such, the main adverse effect of warfarin is hemorrhage, which is not predictable based on the dosage administered. Anticoagulant rodenticides initially cause prolongation of the prothrombin time at 24–36 h post-ingestion. This coincides with a depletion of Factor VII which has the shortest half-life of all the vitamin K-dependent factors (6.2 h in the dog) and whose activity is measured by the PT. (72) Prolongation of the aPTT follows when there is a depletion of other coagulation factors. A 3-year-old Boxer dog has been reported with hematomyelia secondary to warfarin ingestion causing non-ambulatory tetraparesis (73). Extradural hemorrhage has also been reported in a dog exposed to diphacinone (74).

- Snake envenomation

A dog has been described with coagulopathy secondary to a brown snake (Pseudonaja species) which experienced paraparesis within 12 h and which progressed over the next 2 days (75). An extradural hematoma was removed surgically and the dog recovered with minimal complications. Although cerebral hemorrhage has been described, secondary to snake bites, the authors could not find evidence of human or veterinary cases of hematomyelia.

- Vasculopathy - there are multiple non-traumatic conditions that affect the structural integrity of the vascular supply to the cord:
  - Vascular malformations

CNS vascular lesions have been categorized into three major groups: (i) reactive vascular proliferations, (ii) vascular malformations including benign neoplasms (e.g., hemangioma) and hamartomas, and (iii) neoplastic disorders (e.g., hemangioblastoma, hemangiosarcoma) (13). The most widely accepted classification system of CNS vascular malformations in people separates lesions into arteriovenous malformations (AVMs), cavernous malformations (CVMs), venous malformations, and capillary telangiectasia (76); a similar classification appears to exist in veterinary medicine (77). Venous malformations and capillary telangiectasia do not carry a significant risk for acute hemorrhage, unlike AVMs and CVMs. Spinal cord AVMs in people can be further divided into dural arteriovenous fistulae and intradural AVMs, which include glomus, juvenile, and fistulous subtypes (78).

Very few reports of vascular malformations exist in veterinary medicine but there have been descriptions of associated hemorrhage causing clinical signs, their imaging characteristics and surgical resection (79–87). Specifically, intramedullary cavernous malformations were described in 2 dogs with myelography identifying an intramedullary lesion proven to be extensive intraparenchymal hemorrhage on postmortem associated with a distinct lobulated intramedullary mass (79); 2 other dogs with intramedullary hemangiomas have had their MRI characteristics described confirming hyperintensity on both T1W and T2W sequences (82); an intramedullary hemangioma was excised via a myelotomy in a 5 ½-year-old Leonberger and an undefined vascular malformation was removed similarly from a 3 ½-year-old cross-breed dog and a 1-year-old Labrador retriever, with long term remission achieved in all 3 (80, 83, 84);

- Vasculitis

Vasculitis refers to inflammation of the blood vessel wall that may develop without apparent cause (primary vasculitis), or in response to a range of initiating insults (secondary vasculitis) (88). There are few defined syndromes in veterinary medicine that are referable to primary vasculitis, but these include the systemic necrotizing polyarteritis syndrome described in colonies of laboratory Beagle dogs, now identified clinically in many breeds and referred to as steroid-responsive meningitis-arteritis (SRMA) (89). SRMA typically results in juvenile dogs experiencing severe neck pain, fever, and an inflammatory leukogram. Dogs with SRMA can experience secondary extradural, intradural-extramedullary or intramedullary hemorrhage (90–93). Spinal hemorrhage has been documented to affect 9.4% of 53 dogs with SRMA and was associated with the presence of paresis/paralysis (93).

Secondary vasculitis has been reported in association with a wide range of infectious, inflammatory, and immune-mediated diseases in animals and humans. Of these, reported causes include autoimmune diseases such as systemic lupus erythematosus (SLE), infection with *Leishmania* spp., *Rickettsia* spp., *Angiostrongylus vasorum*, and *Dirofilaria immitis*, and exposure to therapeutic products such as carprofen and meloxicam (88).

In canine leishmaniasis, immune complexes may be deposited on blood vessels as a consequence of the persistent production of circulating antigens. These soluble complexes activate the complement cascade, which elicits an inflammatory response and may cause systemic (necrotizing) vasculitis (94). A 14-month-old cross-breed dog with paraplegia has been reported with hematomyelia secondary to leishmania and vasculitis (95).

- Hemorrhagic transformation of an ischemic lesion (e.g., fibrocartilaginous myelopathy)

Infarcted areas of the spinal cord secondary to fibrocartilaginous embolic myelopathy are usually ischemic but there can also be a hemorrhagic component (61, 96).

- Radiation therapy

Hematomyelia has been reported secondary to spinal cord radiation therapy (61, 97). Hemorrhage primarily involved the white matter, but also involved one or more of the gray matter horns at different levels of the spinal cord. The ED_50_ for massive cord hemorrhage is 65.8 Gy based on one histopathologic study (97).

- Neoplastic disease

**Figure 10:**
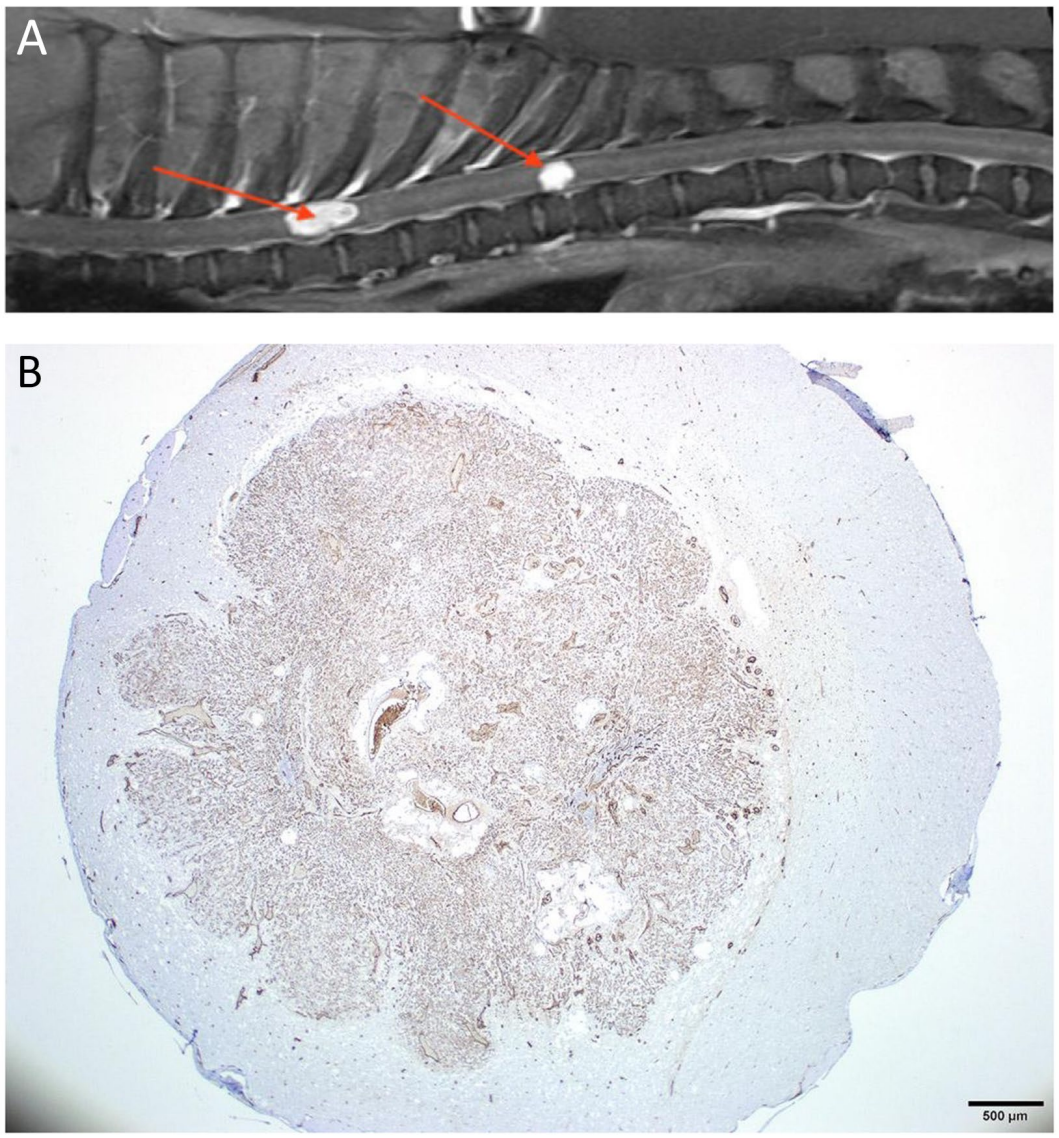
(A) Sagittal T1W post-contrast thoracolumbar vertebral column MRI of a 5-year-old mixed-breed dog with a chronic progression of paraparesis and back pain. There are 2 well-defined markedly contrast-enhancing intramedullary lesions (arrows). (B) A histopathological transverse section of the spinal cord at the level of the caudal lesion seen in A. The section is stained for the expression of factor VIII/von Willebrand factor (FVIII/vWF), a specific marker for endothelial cells and reveals that the intramedullary lesion intensely expressed the stain. The lesion was compatible with a hemangioblastoma.

Hemangioblastoma, astrocytomas, or ependymomas may have intralesional hemorrhage. However, they normally show a solid portion and some degree of contrast enhancement (Figure 10) (98). Extradural hemorrhage has been associated with a primary epidural hemangiosarcoma in a dog (99). The MRI features of both extra-and intramedullary hemangiosarcomas have recently been described, with intramedullary lesions being cervical, metastatic, and frequently accompanied by intracranial lesions (100).

- Idiopathic

**Figure 11:**
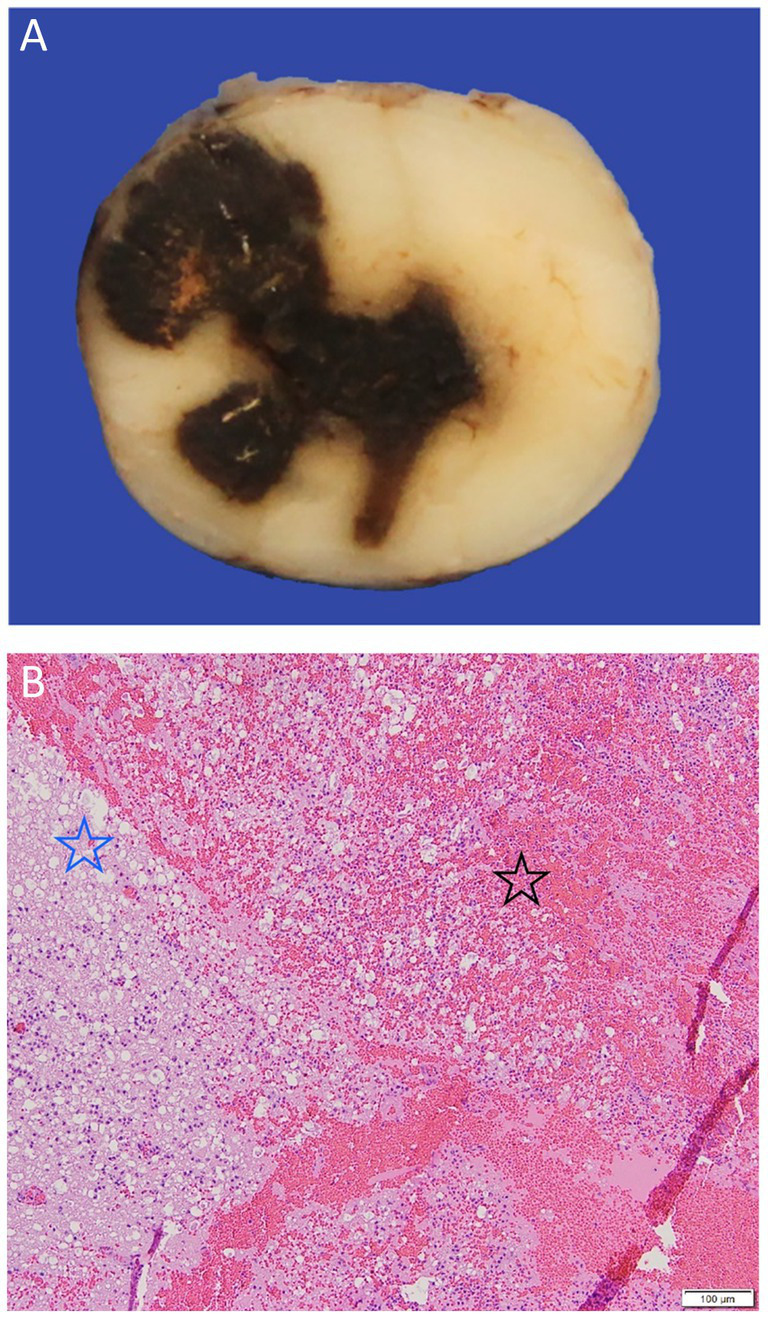
(A) A gross pathological cross-section of the cranial thoracic spinal cord of a 4-year-old French bulldog with acute progressive paraparesis secondary to primary hematomyelia. There is an extensive drak red/black region of hemorrhage seen replacing the neuroparenchyma. (B) An H&E x 5 image of the border of the extensive hemorrhage (black star) seen in A, which can be seen to be effacing and replacing the neuroparenchyma and compressing the adjacent spinal cord tissue (blue star).

When no cause of the hemorrhage can be identified, the etiology is classified as idiopathic which is the most common single classification when considering NTSH (35% of 23 dogs) (61). This has been termed primary hematomyelia (101, 102) (Figure 11).

### Localization

Regardless of species, hemorrhagic myelopathies can be classified based on the location of the bleeding just as they can for encephalopathies, even though this review focuses on intramedullary bleeds. Intraspinal but extramedullary hemorrhage is termed hematorrhachis while hemorrhage within the spinal cord is called hematomyelia. Spinal hematoma location(s) include epidural, subdural, subarachnoid, intramedullary, or a combination of these. This may be further classified based on specific localization along the craniocaudal axis, left–right lateralization, and gray versus white matter, for instance.

In humans, subarachnoid hemorrhage in the spine occurs in 6/100,000 people annually. Approximately 85% of the time, SAH in the spinal column is related to an intracranial aneurysm rupture (45) Spinal epidural hematoma is the next most common spinal hemorrhage in people, although still rare. The incidence of this pathology is approximately 0.1 per 100,000. Spinal EDH is usually caused by epidural venous plexus hemorrhage, which may be caused by minor trauma, coagulopathy, transmitted venous hypertension, or intervertebral disk herniation (103).

In veterinary medicine, although specific localization is difficult to confirm based on imaging studies, spinal hemorrhage may be located in any of the following locations (61):

- Intradural-extramedullary
  - Subarachnoid hemorrhage
  - Subdural hemorrhage
  - Subpial hemorrhage
- Intramedullary
- Extradural

Further anatomical differentiation of intramedullary hemorrhage may focus on segmental localization (i.e., C1-C5, T3-L3, etc.) and whether it can be determined to be affecting white matter, gray matter, or the central canal.

### Clinical signs

Clinical signs related to hemorrhagic myelopathies are primarily dependent on the location of the hemorrhage within the spinal cord tissue, extent of hemorrhage, associated pathology, secondary effects (e.g., mass effect), and time frame (e.g., acute versus chronic phase). Therefore, signs vary, but predominantly include paresis (lower motor neuron when cervical or lumbosacral intumescences are involved), proprioceptive ataxia, spinal reflex deficits, proprioceptive deficits, abnormal postures, hyperesthesia, and abnormal sensation.

The majority of cases of NTSH will have an onset of clinical signs within 72 h and up to 70% can exhibit progressive neurological signs from hours to several weeks (61); however, the median duration of progression is 24 h. Several grading systems have been used to assess the severity of neurological signs in dogs and cats with a spinal cord lesion. These include the Texas Spinal Cord Injury Score and the Modified Frankel Scale (104, 105).

For humans patients with hematomyelia, a clinical triad of local spinal pain, radicular pain, and long-tract signs has been described (106).

### Diagnostic considerations

In cases with suspected TSH, a systemic physical and imaging evaluation should be considered before further evaluation of the vertebral column, with an urgent focus on the evaluation of the patient’s airway, breathing function, circulation, and the possibility of ongoing exsanguination. Imaging assessment of the vertebral column should preferably be performed using CT and or MRI and should not solely focus on the lesion localization determined by a neurological examination in case of multifocal injury.

The most sensitive diagnostic test to confirm the presence of intramedullary hemorrhage is MRI. Differentiating spinal hematoma from inflammatory or neoplastic lesions can be challenging. The clinical history and contrast-enhanced imaging may help to differentiate pathologies, i.e., malignancy such as infiltrative lymphoma or metastasis will enhance although contrast enhancement of intraspinal hemorrhage has been reported in dogs (66, 107). Contrast imaging may identify underlying intramedullary lesions such as hemorrhagic ependymoma which enhance avidly. Additionally, intramedullary hemorrhage is frequently accompanied by cord edema, seen as hyperintensity on T2 weighted sequences, with loss of the gray-white matter differentiation on transverse images. MR imaging has been described in experimental dogs with acute traumatic lesions of various severities which found variation in location and extent of the resultant hemorrhage based on the associated severity of the impact (108). In severe traumatic-impact injuries. MR showed widespread longitudinal extension of the hemorrhage with involvement of the central and periphery of the spinal cord versus a more central location after low-impact injuries.

**Figure 12:**
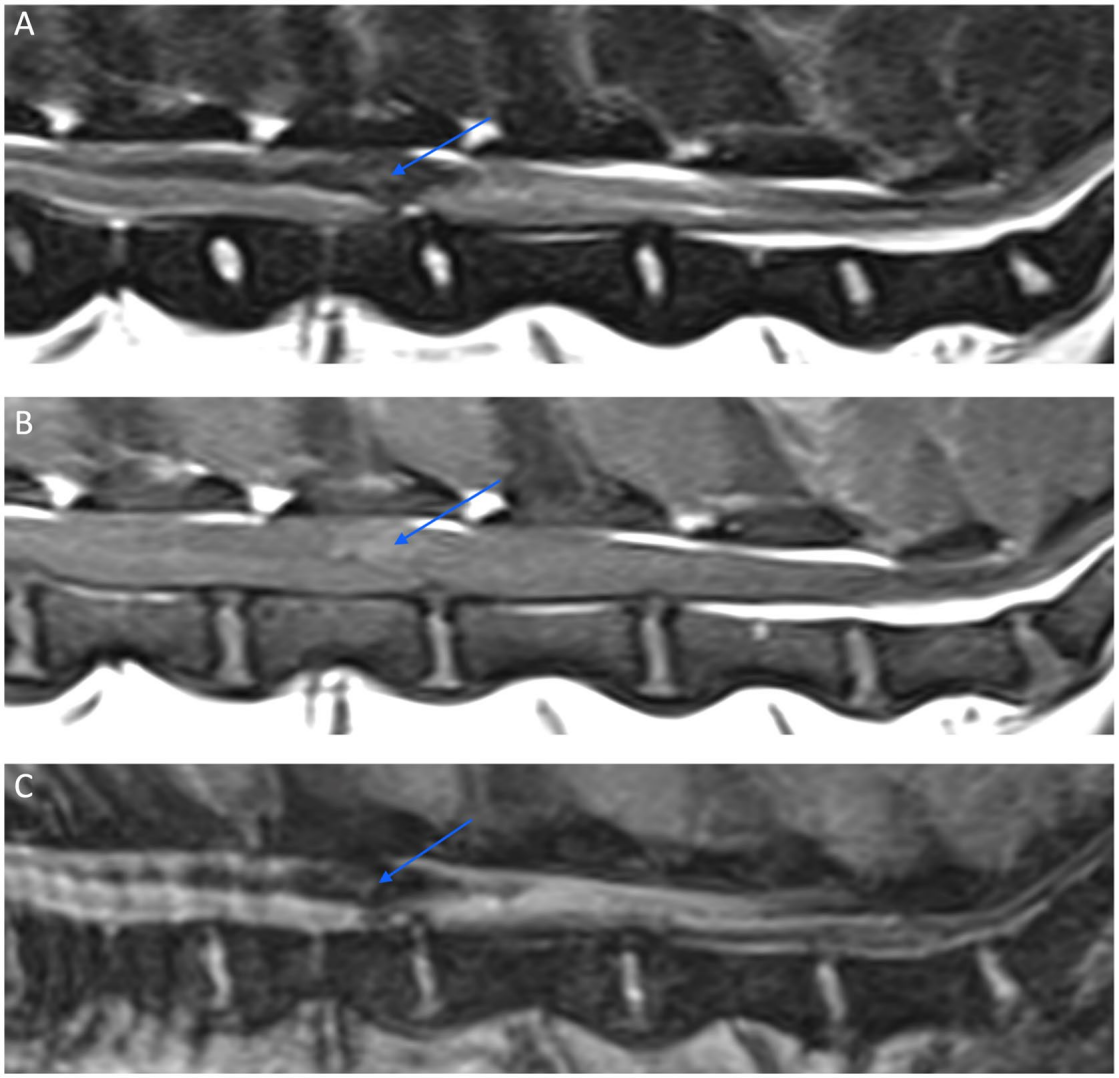
Sagittal MR images of the caudal lumbar vertebral column of a 9-year-old Beagle with suspected primary intramedullary hemorrhage. A relatively well-defined irregular intramedullary hypointense lesion (arrow) is seen on a T2W image (A) extending over the L3 and L4 vertebral bodies. The lesion has a mild T1W pre-contrast hyperintense signal (B), and exhibits a signal void on a Multiple Echo Data Image Combination (MEDIC) (C), which is a T2*-weighted spoiled gradient echo sequence.

As for intracranial hemorrhage, the imaging features of hematomas on MRI evolve over time (109); the progression of hemoglobin degradation and therefore the imaging findings may proceed differently than in the brain due to variability in the local environment, but this has not been established in the dog (107). Due to the presence of intracellular oxyhemoglobin, spinal hematoma in the hyperacute phase appear isointense on T1 and hyperintense on T2 weighted imaging. A rim of hypointensity surrounding the hematoma is variably seen. Intracellular deoxyhemoglobin accounts for acute phase imaging characteristics; T2 signal intensity decreases and hematoma appears hypointense. T1 signal remains intermediate to long and hematoma, therefore appears hypo−/isointense. In the early subacute phase, T1 signal intensity increases, T2 shortens and hematoma appears T1 hyperintense and T2 hypointense (Figure 12). In the late subacute phase, extracellular methemoglobin is formed and hematoma is visualized as both T1 and T2 hyperintense. Paramagnetic hemosiderin and ferritin account for the usual appearance of chronic hematoma which is hypointense on both T1 and T2 weighted imaging. It should be noted that signal voids on spin-echo sequences are associated with gas, cortical bone, calcification, fibrous tissue, metallic implants, fast-flowing blood, and blood-breakdown products (110).

A gradient echo T2-weighted (GRE) sequence is the most sensitive method to detect hemorrhage (111). However, a signal void on GRE sequences may not be seen in hyperacute stages (before conversion to deoxyhemoglobin) or chronic hemorrhage (because of the homogenous distribution of extracellular methemoglobin) (112).

CT of the spine may also be used to identify the hemorrhage and any evidence of associated spine pathology. Spinal cord hemorrhage can present as an expansile short or long segment area of hyperattenuation on CT images, which may be multifocal and may be contrast-enhancing potentially surrounded by a hypoattenuating region of edema (45, 64). However, overall MRI is a more valuable technique for the detection of chronic hemorrhage, which may be invisible on CT (113).

Specific etiology testing should focus on the potential for a bleeding diathesis starting with evaluation for *A. vasorum* using an in-clinic assay for detection of circulating antigen (Angio Detect Test, IDEXX Laboratories, Westbrook, Maine, USA) in addition to looking for the presence of first-stage *A. vasorum* larvae on fecal analysis (Baermann method). Further testing should include coagulation assays [prothrombin time (PT), activated partial thromboplastin time (aPTT), buccal mucosal bleeding time (BMBT)] and if there are abnormalities, they should be followed by platelet quantification and specific coagulation factor testing.

In humans, in addition to standard MR or CT imaging, magnetic resonance angiography is often considered and CT angiography is a valid alternative (46). Angiography involves sequential, multiple injections of potentially parent vessels to identify the vascular pathology. However, locating the source of the hemorrhage by angiography is often a challenge especially when an isolated aneurysm is the culprit. MR and CT spinal angiography has been described and used to evaluate the vasculature of the canine spinal cord (114, 115).

### Treatment considerations

In the absence of clinical trials to guide the treatment of these rare conditions, management is often aimed at underlying causes if known, and supportive care. Conservative treatment may be justified in cases with minimal or rapidly improving neurological deficits and has been documented to be successful in dogs with TSH (49). If TSH is suspected, basic support measures and general patient stabilization is required before focusing on specific treatment measures in order to maintain adequate spinal cord perfusion (116). Maintaining blood pressure within standard reference ranges can be and is regarded as a cornerstone of treatment for this aim (117).

However, there does not appear to be a consensus on whether medical or surgical treatment is.

more appropriate and it is currently suggested that conservative management may be reserved for cases with mild neurological manifestation or increased bleeding tendency, whereas surgery is a viable option when the neurological signs are severe and progressive, and when removal of the expanding hemorrhagic lesion may be beneficial (44).

**Figure 13:**
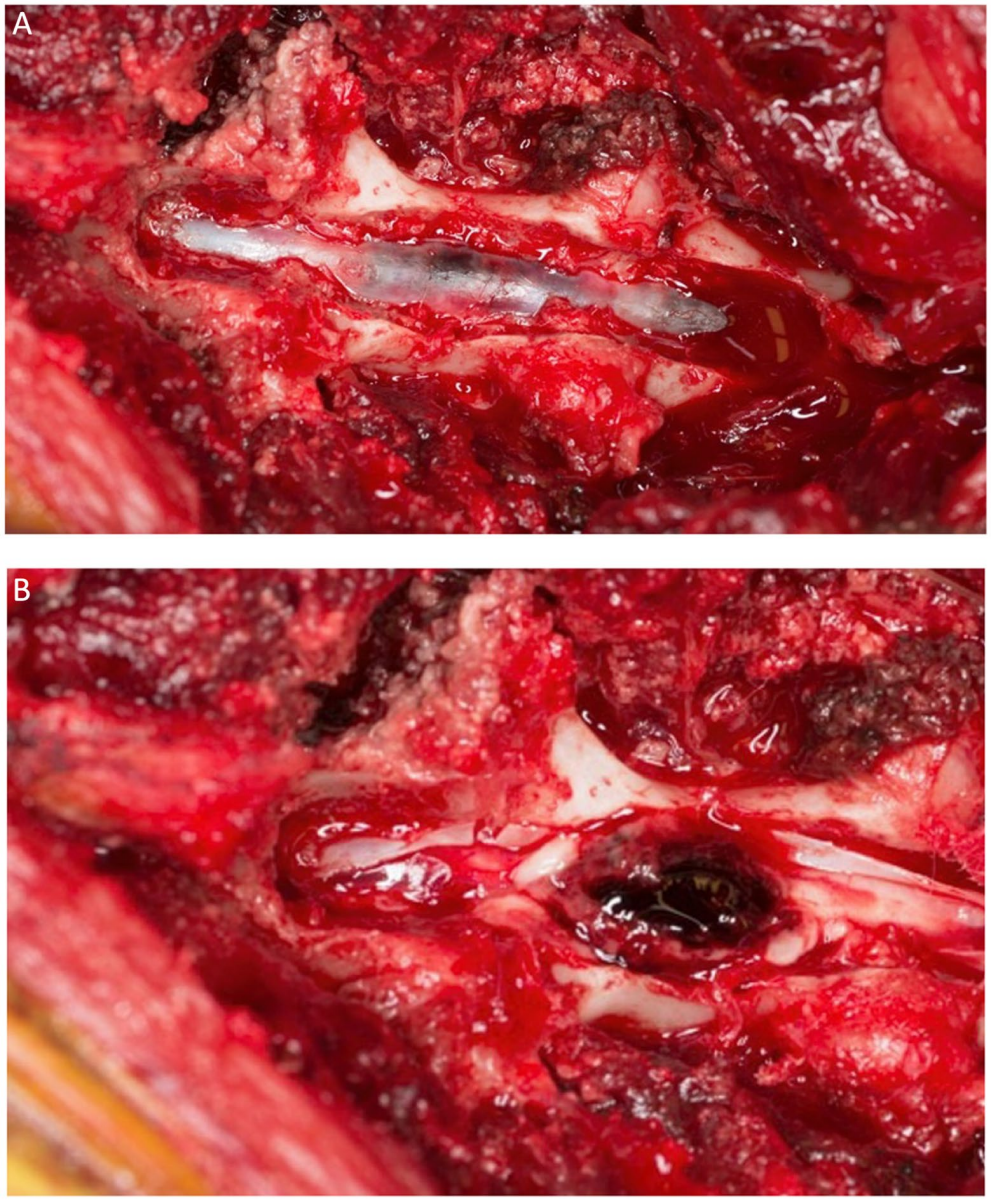
A dorsal view of the spinal cord of the dog of Figure 12 after a dorsal laminectomy (A) and a durotomy (B) revealing a hematoma on the dorsal midline of the cord just prior to its removal.

In humans, management of the acute case is focused on relieving pressure on the spinal cord where individual reports suggest that surgical decompression should be performed as soon as possible to minimize the neurological injury (46). Durotomy and duraplasty have been studied in humans with SCIWORA as a mechanism to indirectly relieve intraspinal pressure (118). Myelotomy has also been described in animal models of SCI and is sporadically reported with positive effects in humans (119). The specific surgical management of intra-medullary hemorrhage focuses on evacuation of the mass effect and parenchymal decompression, minimizing disruption of normal spinal cord parenchyma (Figure 13). Intramedullary hemorrhage, which extends to the surface of the pial spinal cord may establish a safe microsurgical corridor of entry. Successful decompressive surgery of an intramedullary hematoma via myelotomies has previously been described in several dogs and a cat with various underlying etiologies (44, 58, 77, 101).

### Prognostic considerations

In veterinary medicine, outcomes with NTSH have been investigated relative to the underlying cause, MRI findings, lesion localization, and severity of neurological signs. No association was found between outcome and the presence of an underlying cause when grouping dogs with underlying causes of NTSH together and comparing to the idiopathic group in a study of 23 dogs (61). However, underlying causes associated with a poor outcome include radiotherapy-induced hemorrhage, acute lymphoid leukemia, FCE, and hemophilia A.

In relation to MRI findings, a study of 23 dogs with NTSH found that the mean length of the hemorrhagic lesion was 4 times higher and the mean length of spinal cord edema was 2 times higher in dogs with a poor outcome compared to dogs with an excellent outcome (61). However, these findings were not significant. Based on this same small study, and unusually, no significant association was found between outcome and severity of neurological signs at initial presentation. A study of 82 dogs presenting with paraplegia and absent nociception secondary to disk extrusion demonstrated that only 33% regained nociception if MRI revealed intramedullary gradient echo signal voids, compared to 67% that recovered and did not have such voids (120).

Outcome associated with TSH is related to clinical signs at the outset. For dogs with disk extrusion, the extent of intramedullary hemorrhage is significantly associated with the severity of spinal cord destruction at the site of the herniation (56). Additionally, the degree of intramedullary hemorrhage is significantly associated with the rostrocaudal extension of myelomalacia suggesting that hematomyelia may contribute to the further progression of myelomalacia. Hemorrhage within the central canal in spinal segments cranial and caudal to the disk extrusion is a finding strongly associated with high grades of tissue destruction and in one study was seen in 75% of dogs with ascending-descending myelomalacia (56).

In human patients with hemorrhagic myelopathy, no association has been found between patient outcome and the location or extent of the hemorrhagic lesion on MRI. However, patients with more severe neurological signs, such as complete loss of motor function, loss of nociception, urinary or fecal incontinence, or both, have been reported to have a significantly worse outcome (121).

## Concluding remarks

As hemorrhagic encephalopathies and hematomyelia are more frequently diagnosed in veterinary patients through advanced imaging studies correlated to clinical presentation, classification according to etiology becomes more important to investigate whether certain categories are associated with poor or good outcomes, require surgical intervention, or have implications for diagnostic procedures. Classification systems have been employed for human hemorrhagic encephalopathies and myelopathies and were discussed in this review as background for the proposal of a veterinary classification. The human literature is much more extensive on this topic and thus forms a substantial part of the reference list for this veterinary review. The proposed classifications in this review can be implemented for clinical use and both retrospective and prospective studies.

## References

[1] Meretoja A Strbian D Putaala J Curtze S Haapaniemi E Mustanoja S. SMASH-U: a proposal for etiologic classification of intracerebral hemorrhage. Stroke. (2012) 43:2592–7. doi: 10.1161/STROKEAHA.112.661603, PMID: 22858729

[2] Marti-Fabregas J Prats-Sanchez L Martinez-Domeno A Camps-Renom P Marin R Jimenez-Xarrie E. The H-ATOMIC criteria for the etiologic classification of patients with intracerebral hemorrhage. PLoS One. (2016) 11:e0156992. doi: 10.1371/journal.pone.0156992, PMID: 27275863 PMC4898692

[3] Raposo N Zanon Zotin MC Seiffge DJ Li Q Goeldlin MB Charidimou A. A causal classification system for intracerebral hemorrhage subtypes. Ann Neurol. (2023) 93:16–28. doi: 10.1002/ana.26519, PMID: 36197294 PMC9839566

[4] Rodrigues LL Mesquita LP Costa RC Gomes RG Biihrer DA Maiorka PC. Multiple infarcts and hemorrhages in the central nervous system of a dog with cerebral amyloid angiopathy: a case report. BMC Vet Res. (2018) 14:370. doi: 10.1186/s12917-018-1700-0, PMID: 30482198 PMC6258392

[5] Bano S Yadav SN Garga UC Chaudhary V. Sporadic cerebral amyloid angiopathy: an important cause of cerebral hemorrhage in the elderly. J Neurosci Rural Pract. (2011) 2:87–91. doi: 10.4103/0976-3147.80107, PMID: 21716867 PMC3122986

[6] Borras D Ferrer I Pumarola M. Age-related changes in the brain of the dog. Vet Pathol. (1999) 36:202–11. doi: 10.1354/vp.36-3-202 10332828

[7] Dewey CW Rishniw M Johnson PJ Davies ES Sackman JJ O'Donnell M. Interthalamic adhesion size in aging dogs with presumptive spontaneous brain microhemorrhages: a comparative retrospective MRI study of dogs with and without evidence of canine cognitive dysfunction. Peer J. (2020) 8:e9012. doi: 10.7717/peerj.9012, PMID: 32322448 PMC7161569

[8] Kerwin SC Levine JM Budke CM Griffin JFT Boudreau CE. Putative cerebral microbleeds in dogs undergoing magnetic resonance imaging of the head: a retrospective study of demographics, clinical associations, and relationship to case outcome. J Vet Intern Med. (2017) 31:1140–8. doi: 10.1111/jvim.14730, PMID: 28556471 PMC5508348

[9] Haller S Montandon ML Lazeyras F Scheffler M Meckel S Herrmann FR. Radiologic-histopathologic correlation of cerebral microbleeds using pre-mortem and post-mortem MRI. PLoS One. (2016) 11:e0167743. doi: 10.1371/journal.pone.0167743, PMID: 27936213 PMC5147972

[10] Shoamanesh A Kwok CS Benavente O. Cerebral microbleeds: histopathological correlation of neuroimaging. Cerebrovasc Dis. (2011) 32:528–34. doi: 10.1159/000331466, PMID: 22104448

[11] Arnold SA Platt SR Gendron KP West FD. Imaging ischemic and hemorrhagic disease of the brain in dogs. Front Vet Sci. (2020) 7:279. doi: 10.3389/fvets.2020.00279 32528985 PMC7266937

[12] Boudreau CE. An update on cerebrovascular disease in dogs and cats. Vet Clin North Am Small Anim Pract. (2018) 48:45–62. doi: 10.1016/j.cvsm.2017.08.009, PMID: 29056397

[13] Marr J Miranda IC Miller AD Summers BA. A review of proliferative vascular disorders of the central nervous system of animals. Vet Pathol. (2021) 58:864–80. doi: 10.1177/0300985820980707 33302811

[14] Dai J Li S Li X Xiong W Qiu Y. The mechanism of pathological changes of intraventricular hemorrhage in dogs. Neurol India. (2009) 57:567–77. doi: 10.4103/0028-3886.57798 19934554

[15] Bhatt SH Kodankandath TV. Subpial hemorrhage in an adult male. Cureus. (2022) 14:e28404. doi: 10.7759/cureus.28404 36171846 PMC9509003

[16] Barreto ARF Carrasco M Dabrowski AK Sun LR Tekes A. Subpial hemorrhage in neonates: what radiologists need to know. AJR Am J Roentgenol. (2021) 216:1056–65. doi: 10.2214/AJR.20.22999, PMID: 33566637

[17] Leech RW Kohnen P. Subependymal and intraventricular hemorrhages in the newborn. Am J Pathol. (1974) 77:465–75. PMID: 4473900 PMC1910930

[18] Roth P Happold C Eisele G Nagele T Weller M Luft AR. A series of patients with subpial hemorrhage: clinical manifestation, neuroradiological presentation and therapeutic implications. J Neurol. (2008) 255:1018–22. doi: 10.1007/s00415-008-0824-8, PMID: 18458859

[19] Caceres JA Goldstein JN. Intracranial hemorrhage. Emerg Med Clin North Am. (2012) 30:771–94. doi: 10.1016/j.emc.2012.06.003, PMID: 22974648 PMC3443867

[20] Yanai H Tapia-Nieto R Cherubini GB Caine A. Results of magnetic resonance imaging performed within 48 hours after head trauma in dogs and association with outcome: 18 cases (2007-2012). J Am Vet Med Assoc. (2015) 246:1222–9. doi: 10.2460/javma.246.11.1222, PMID: 25970219

[21] Hayman LA McArdle CB Taber KH Saleem A Baskin D Lee HS. MR imaging of hyperacute intracranial hemorrhage in the cat. AJNR Am J Neuroradiol. (1989) 10:681–6. 2505498 PMC8332628

[22] Kuo KW Bacek LM Taylor AR. Head Trauma. Vet Clin North Am Small Anim Pract. (2018) 48:111–28. doi: 10.1016/j.cvsm.2017.08.005 28985897

[23] Hecht S Anderson KM Castel A JFT G Hespel AM Nelson N. Agreement of magnetic resonance imaging with computed tomography in the assessment for acute skull fractures in a canine and feline cadaver model. Front Vet Sci. (2021) 8:603775. doi: 10.3389/fvets.2021.603775, PMID: 33969028 PMC8100023

[24] Beltran E Platt SR McConnell JF Dennis R Keys DA De Risio L. Prognostic value of early magnetic resonance imaging in dogs after traumatic brain injury: 50 cases. J Vet Intern Med. (2014) 28:1256–62. doi: 10.1111/jvim.12368, PMID: 24814522 PMC4857941

[25] Hostettler IC Seiffge DJ Werring DJ. Intracerebral hemorrhage: an update on diagnosis and treatment. Expert Rev Neurother. (2019) 19:679–94. doi: 10.1080/14737175.2019.1623671 31188036

[26] Goeldlin M Stewart C Radojewski P Wiest R Seiffge D Werring DJ. Clinical neuroimaging in intracerebral haemorrhage related to cerebral small vessel disease: contemporary practice and emerging concepts. Expert Rev Neurother. (2022) 22:579–94. doi: 10.1080/14737175.2022.2104157, PMID: 35850578

[27] Sande A West C. Traumatic brain injury: a review of pathophysiology and management. J Vet Emerg Crit Care (San Antonio). (2010) 20:177–90. doi: 10.1111/j.1476-4431.2010.00527.x 20487246

[28] Cordonnier C Demchuk A Ziai W Anderson CS. Intracerebral haemorrhage: current approaches to acute management. Lancet. (2018) 392:1257–68. doi: 10.1016/S0140-6736(18)31878-6 30319113

[29] Greenberg SM Ziai WC Cordonnier C Dowlatshahi D Francis B Goldstein JN. 2022 guideline for the Management of Patients with Spontaneous Intracerebral Hemorrhage: a guideline from the American Heart Association/American Stroke Association. Stroke. (2022) 53:e282–361. doi: 10.1161/STR.0000000000000407, PMID: 35579034

[30] Platt SR Radaelli ST McDonnell JJ. The prognostic value of the modified Glasgow coma scale in head trauma in dogs. J Vet Intern Med. (2001) 15:581–4. doi: 10.1111/j.1939-1676.2001.tb01594.x, PMID: 11817064

[31] Cameron S Weltman JG Fletcher DJ. The prognostic value of admission point-of-care testing and modified Glasgow coma scale score in dogs and cats with traumatic brain injuries (2007-2010): 212 cases. J Vet Emerg Crit Care (San Antonio). (2022) 32:75–82. doi: 10.1111/vec.13108 34432934

[32] Lowrie M De Risio L Dennis R Llabres-Diaz F Garosi L. Concurrent medical conditions and long-term outcome in dogs with nontraumatic intracranial hemorrhage. Vet Radiol Ultrasound. (2012) 53:381–8. doi: 10.1111/j.1740-8261.2012.01934.x, PMID: 22537251

[33] Garosi LS. Cerebrovascular disease in dogs and cats. Vet Clin North Am Small Anim Pract. (2010) 40:65–79. doi: 10.1016/j.cvsm.2009.09.001 19942057

[34] Garosi LS Platt SR McConnell JF Wrayt JD Smith KC. Intracranial haemorrhage associated with Angiostrongylus vasorum infection in three dogs. J Small Anim Pract. (2005) 46:93–9. doi: 10.1111/j.1748-5827.2005.tb00300.x, PMID: 15736817

[35] Wyatt S Llabres-Diaz F Lee CY Beltran E. Early CT in dogs following traumatic brain injury has limited value in predicting short-term prognosis. Vet Radiol Ultrasound. (2021) 62:181–9. doi: 10.1111/vru.12933, PMID: 33241888

[36] Mann O Peery D Bader Segev R Klainbart S Kelmer E Sobarzo A. CT findings and the prognostic value of the Koret CT score in cats with traumatic brain injury. J Feline Med Surg. (2022) 24:91–7. doi: 10.1177/1098612X211005306, PMID: 33847537 PMC8807991

[37] Caine A Brash R De Risio L Van Dijk J Cherubini GB Dennis R. MRI in 30 cats with traumatic brain injury. J Feline Med Surg. (2019) 21:1111–9. doi: 10.1177/1098612X18819162, PMID: 30565962 PMC10814270

[38] Prats-Sanchez L Iruzubieta P Vesperinas A Collet R Martinez-Domeno A Guisado-Alonso D. Frequency, predictors, etiology, and outcomes for deep intracerebral hemorrhage without hypertension. J Stroke Cerebrovasc Dis. (2022) 31:106293. doi: 10.1016/j.jstrokecerebrovasdis.2021.106293, PMID: 35016096

[39] Malhotra K Zompola C Theodorou A Katsanos AH Shoamanesh A Gupta H. Prevalence, characteristics, and outcomes of undetermined intracerebral hemorrhage: a systematic review and Meta-analysis. Stroke. (2021) 52:3602–12. doi: 10.1161/STROKEAHA.120.031471, PMID: 34344165

[40] Lei C Wu B Liu M Tan G Zeng Q. Pathogenesis and subtype of intracerebral hemorrhage (ICH) and ICH score determines prognosis. Curr Neurovasc Res. (2016) 13:244–8. doi: 10.2174/1567202613666160527141128, PMID: 27229323

[41] Ruiz-Sandoval JL Cantu C Barinagarrementeria F. Intracerebral hemorrhage in young people: analysis of risk factors, location, causes, and prognosis. Stroke. (1999) 30:537–41. doi: 10.1161/01.STR.30.3.537 10066848

[42] Karavelis A Foroglou G Petsanas A Zarampoukas T. Spinal cord dysfunction caused by non-traumatic hematomyelia. Spinal Cord. (1996) 34:268–71. doi: 10.1038/sc.1996.48, PMID: 8963973

[43] Atesok K Tanaka N O'Brien A Robinson Y Pang D Deinlein D. Posttraumatic spinal cord injury without radiographic abnormality. Adv Orthop. (2018) 2018:1–10. doi: 10.1155/2018/7060654 PMC5817293 29535875

[44] Guo S. Surgical treatment and outcome of haematomyelia with a traumatic cause in a dog and a cat. Vet. Med Sci. (2024) 10:e 1377. doi: 10.1002/vms3.1377 PMC10867873 38358058

[45] Vuong SM Jeong WJ Morales H Abruzzo TA. Vascular diseases of the spinal cord: infarction, hemorrhage, and venous congestive myelopathy. Semin Ultrasound CT MR. (2016) 37:466–81. doi: 10.1053/j.sult.2016.05.008, PMID: 27616317

[46] Shaban A Moritani T Al Kasab S Sheharyar A Limaye KS Adams HP Jr. Spinal cord hemorrhage. J Stroke Cerebrovasc Dis. (2018) 27:1435–46. doi: 10.1016/j.jstrokecerebrovasdis.2018.02.014 29555403

[47] Agarwal A Kanekar S Thamburaj K Vijay K. Radiation-induced spinal cord hemorrhage (hematomyelia). Neurol Int. (2014) 6:5553. doi: 10.4081/ni.2014.5553, PMID: 25568739 PMC4274409

[48] Da Ros V Picchi E Ferrazzoli V Schirinzi T Sabuzi F Grillo P. Spinal vascular lesions: anatomy, imaging techniques and treatment. Eur J Radiol Open. (2021) 8:100369. doi: 10.1016/j.ejro.2021.100369, PMID: 34307789 PMC8283341

[49] Santifort KM Carrera I Platt S. Case report: traumatic hemorrhagic cervical myelopathy in a dog. Front Vet Sci. (2023) 10:1260719. doi: 10.3389/fvets.2023.1260719, PMID: 37869493 PMC10585029

[50] Merbl Y Valerio-Lopez C. What is your neurologic diagnosis? J Am Vet Med Assoc. (2022) 260:1–4. doi: 10.2460/javma.21.01.0052 35263292

[51] Castel A Olby NJ Mariani CL Munana KR Early PJ. Clinical characteristics of dogs with progressive Myelomalacia following acute intervertebral disc extrusion. J Vet Intern Med. (2017) 31:1782–9. doi: 10.1111/jvim.14829, PMID: 28961348 PMC5697170

[52] De Risio L. A review of Fibrocartilaginous embolic myelopathy and different types of Peracute non-compressive intervertebral disk extrusions in dogs and cats. Front Vet Sci. (2015) 2:24. doi: 10.3389/fvets.2015.00024 26664953 PMC4672181

[53] Tartarelli CL Baroni M Borghi M. Thoracolumbar disc extrusion associated with extensive epidural haemorrhage: a retrospective study of 23 dogs. J Small Anim Pract. (2005) 46:485–90. doi: 10.1111/j.1748-5827.2005.tb00277.x, PMID: 16245662

[54] Fenn J Olby NJ. Canine spinal cord injury C. Classification of intervertebral disc disease. Front Vet Sci. (2020) 7:579025. doi: 10.3389/fvets.2020.579025, PMID: 33134360 PMC7572860

[55] Losey P Young C Krimholtz E Bordet R Anthony DC. The role of hemorrhage following spinal-cord injury. Brain Res. (2014) 1569:9–18. doi: 10.1016/j.brainres.2014.04.033 24792308

[56] Henke D Gorgas D Doherr MG Howard J Forterre F Vandevelde M. Longitudinal extension of myelomalacia by intramedullary and subdural hemorrhage in a canine model of spinal cord injury. Spine J. (2016) 16:82–90. doi: 10.1016/j.spinee.2015.09.018, PMID: 26386168

[57] Kent M Eagleson JS Neravanda D Schatzberg SJ Gruenenfelder FI Platt SR. Intraaxial spinal cord hemorrhage secondary to atlantoaxial subluxation in a dog. J Am Anim Hosp Assoc. (2010) 46:132–7. doi: 10.5326/0460132, PMID: 20194370

[58] Platt SR Dennis R Murphy K De Stefani A. Hematomyelia secondary to lumbar cerebrospinal fluid acquisition in a dog. Vet Radiol Ultrasound. (2005) 46:467–71. doi: 10.1111/j.1740-8261.2005.00085.x 16396261

[59] Dutil GF Schweizer D Oevermann A Stein VM Maiolini A. Haematomyelia and myelomalacia following an inadvertent thoracic intraspinal injection in a cat. JFMS Open Rep. (2021) 7:205511692199539. doi: 10.1177/2055116921995394 PMC7968032 33796326

[60] Kishimoto M Yamada K Ueno H Kobayashi Y Wisner ER. Spinal cord effects from lumbar myelographic injection technique in the dog. J Vet Med Sci. (2004) 66:67–9. doi: 10.1292/jvms.66.67, PMID: 14960814

[61] West N Butterfield S Rusbridge C Fernandez A Tabanez J Rudolf NJ. Non-traumatic hemorrhagic myelopathy in dogs. J Vet Intern Med. (2023) 37:1129–38. doi: 10.1111/jvim.16694, PMID: 37095733 PMC10229319

[62] Brooks M. A review of canine inherited bleeding disorders: biochemical and molecular strategies for disease characterization and carrier detection. J Hered. (1999) 90:112–8. doi: 10.1093/jhered/90.1.112, PMID: 9987916

[63] Barr JW McMichael M. Inherited disorders of hemostasis in dogs and cats. Top Companion Anim Med. (2012) 27:53–8. doi: 10.1053/j.tcam.2012.07.006, PMID: 23031456

[64] Barnard LR Leblond G Nykamp SG Gaitero L. Spontaneous thoracolumbar hematomyelia secondary to hemophilia B in a cat. JFMS Open Rep. (2015) 1:2055116915597239. doi: 10.1177/2055116915597239, PMID: 28491378 PMC5362005

[65] Thompson MS Kreeger JM. Acute paraplegia in a puppy with hemophilia a. J Am Anim Hosp Assoc. (1999) 35:36–7. doi: 10.5326/15473317-35-1-36, PMID: 9934926

[66] Fowler KM Bolton TA Rossmeisl JH Arendse AU Vernau KM Li RHL. Clinical, diagnostic, and imaging findings in three juvenile dogs with Paraspinal hyperesthesia or myelopathy as a consequence of hemophilia a: a case report. Front Vet Sci. (2022) 9:871029. doi: 10.3389/fvets.2022.871029, PMID: 35498741 PMC9051508

[67] Lubbers C Beukers M Bergknut N Paes G. Hemophilia a resulting in severe hyperesthesia due to Extraparenchymal spinal cord hemorrhage in a Young Golden retriever puppy. Vet Sci. (2022) 9:638. doi: 10.3390/vetsci9110638 36423087 PMC9697390

[68] Eyster ME Gill FM Blatt PM Hilgartner MW Ballard JO Kinney TR. Central nervous system bleeding in hemophiliacs. Blood. (1978) 51:1179–88. doi: 10.1182/blood.V51.6.1179.1179, PMID: 647123

[69] Guevar J Gutierrez-Quintana R Leach JD Penderis J. What is your neurologic diagnosis? Suspected immune-mediated thrombocytopenia with secondary spinal cord hemorrhage. J Am Vet Med Assoc. (2015) 247:479–82. doi: 10.2460/javma.247.5.479 26295550

[70] Wessmann A Lu D Lamb CR Smyth B Mantis P Chandler K. Brain and spinal cord haemorrhages associated with Angiostrongylus vasorum infection in four dogs. Vet Rec. (2006) 158:858–63. doi: 10.1136/vr.158.25.858, PMID: 16798954

[71] Schelling CG Greene CE Prestwood AK Tsang VC. Coagulation abnormalities associated with acute Angiostrongylus vasorum infection in dogs. Am J Vet Res. (1986) 47:2669–73. PMID: 3800129

[72] Ren P Stark PY Johnson RL Bell RG. Mechanism of action of anticoagulants: correlation between the inhibition of prothrombin synthesis and the regeneration of vitamin K1 from vitamin K1 epoxide. J Pharmacol Exp Ther. (1977) 201:541–6. PMID: 864593

[73] Blanco C Moral M Minguez JJ Lorenzo V. Presumptive Haematomyelia secondary to warfarin Toxicosis in a dog. Case Rep Vet Med. (2022) 2022:1–7. doi: 10.1155/2022/8349085 PMC9371806 35967597

[74] Solari FP Sherman AH Blong AE Cameron S Walton RA. Diagnosis and successful management of an extradural compressive hematoma secondary to diphacinone poisoning in a dog. J Vet Emerg Crit Care (San Antonio). (2023) 33:101–6. doi: 10.1111/vec.13248 36098050 PMC10087660

[75] Ong RK Lenard ZM Swindells KL Raisis AL. Extradural haematoma secondary to brown snake (Pseudonaja species) envenomation. Aust Vet J. (2009) 87:152–6. doi: 10.1111/j.1751-0813.2009.00410.x, PMID: 19335471

[76] Zabramski JM Henn JS Coons S. Pathology of cerebral vascular malformations. Neurosurg Clin N Am. (1999) 10:395–410. doi: 10.1016/S1042-3680(18)30174-8 10419567

[77] Thibaud JL Hidalgo A Benchekroun G Fanchon L Crespeau F Delisle F. Progressive myelopathy due to a spontaneous intramedullary hematoma in a dog: pre-and postoperative clinical and magnetic resonance imaging follow-up. J Am Anim Hosp Assoc. (2008) 44:266–75. doi: 10.5326/0440266, PMID: 18762564

[78] Muraszko KM Oldfield EH. Vascular malformations of the spinal cord and dura. Neurosurg Clin N Am. (1990) 1:631–52. doi: 10.1016/S1042-3680(18)30794-0 2136162

[79] Mac Killop E Olby NJ Linder KE Brown TT. Intramedullary cavernous malformation of the spinal cord in two dogs. Vet Pathol. (2007) 44:528–32. doi: 10.1354/vp.44-4-528, PMID: 17606517

[80] Yanke AB Miller MA Fulkerson CV Bohn K Bentley RT. Remission after complete excision of an intramedullary hemangioma with an identifiable tumor plane in a dog. Vet Surg. (2019) 48:1507–13. doi: 10.1111/vsu.13238, PMID: 31179565

[81] Truong Faulds T Milan V Sharifi-Amina S Stout C Bui D. Intramedullary spinal cord hemangioma: a rare case report. Radiol Case Rep. (2024) 19:223–6. doi: 10.1016/j.radcr.2023.10.006, PMID: 38028300 PMC10630760

[82] Jull P Walmsley GL Benigni L Wenzlow N Rayner EL Summers BA. Imaging diagnosis—spinal cord hemangioma in two dogs. Vet Radiol Ultrasound. (2011) 52:653–7. doi: 10.1111/j.1740-8261.2011.01851.x, PMID: 21831248

[83] Santifort KM Plonek M Grinwis GCM Carrera I Platt S. Case report: surgical treatment and long-term successful outcome of a spinal intramedullary vascular malformation in a dog. Front Vet Sci. (2023) 10:1243882. doi: 10.3389/fvets.2023.1243882, PMID: 37645678 PMC10461059

[84] Alexander K Huneault L Foster R d'Anjou MA. Magnetic resonance imaging and marsupialization of a hemorrhagic intramedullary vascular anomaly in the cervical portion of the spinal cord of a dog. J Am Vet Med Assoc. (2008) 232:399–404. doi: 10.2460/javma.232.3.399, PMID: 18241107

[85] Cordy DR. Vascular malformations and hemangiomas of the canine spinal cord. Vet Pathol. (1979) 16:275–82. doi: 10.1177/030098587901600301, PMID: 442458

[86] Hayashida E Ochiai K Kadosawa T Kimura T Umemura T. Arteriovenous malformation of the cervical spinal cord in a dog. J Comp Pathol. (1999) 121:71–6. doi: 10.1053/jcpa.1998.0294, PMID: 10373295

[87] Zaki FA. Vascular malformation (cavernous angioma) of the spinal cord in a dog. J Small Anim Pract. (1979) 20:417–22. doi: 10.1111/j.1748-5827.1979.tb06746.x 470353

[88] Swann JW Priestnall SL Dawson C Chang YM Garden OA. Histologic and clinical features of primary and secondary vasculitis: a retrospective study of 42 dogs (2004-2011). J Vet Diagn Invest. (2015) 27:489–96. doi: 10.1177/1040638715587934 26077546

[89] Hoff EJ Vandevelde M. Necrotizing vasculitis in the central nervous systems of two dogs. Vet Pathol. (1981) 18:219–23. doi: 10.1177/030098588101800209, PMID: 7467081

[90] Caswell JL Nykamp SG. Intradural vasculitis and hemorrhage in full sibling welsh springer spaniels. Can Vet J. (2003) 44:137–9. 12650042 PMC340048

[91] Hughes KL Stieger-Vanegas SM Valentine BA. Hemorrhage in the central canal of the cervical spinal cord in a coonhound diagnosed with canine juvenile polyarteritis (steroid responsive meningitis-arteritis). Can Vet J. (2015) 56:567–70. 26028675 PMC4431151

[92] Wang-Leandro A Huenerfauth EI Heissl K Tipold A. MRI findings of Early-stage Hyperacute hemorrhage causing Extramedullary compression of the cervical spinal cord in a dog with suspected steroid-responsive meningitis-arteritis. Front Vet Sci. (2017) 4:161. doi: 10.3389/fvets.2017.00161, PMID: 29021984 PMC5623665

[93] Jones BA Agthe P Scarpante E Crawford A Black V Espadas I. Magnetic resonance imaging findings in dogs with steroid-responsive meningitis-arteritis in the UK and their clinical significance: 53 cases (2013-2021). J Small Anim Pract. (2024) 2024:13775. doi: 10.1111/jsap.13775, PMID: 39228252 PMC11736089

[94] Pumarola M Brevik L Badiola J Vargas A Domingo M Ferrer L. Canine leishmaniasis associated with systemic vasculitis in two dogs. J Comp Pathol. (1991) 105:279–86. doi: 10.1016/S0021-9975(08)80196-X, PMID: 1684801

[95] Font A Mascort J Altimira J Closa JM Vilafranca M. Acute paraplegia associated with vasculitis in a dog with leishmaniasis. J Small Anim Pract. (2004) 45:199–201. doi: 10.1111/j.1748-5827.2004.tb00224.x, PMID: 15116888

[96] Cauzinille L. Fibrocartilaginous embolism in dogs. Vet Clin North Am Small Anim Pract. (2000) 30:155–67, vii. doi: 10.1016/S0195-5616(00)50007-2 10680213

[97] Powers BE Beck ER Gillette EL Gould DH LeCouter RA. Pathology of radiation injury to the canine spinal cord. Int J Radiat Oncol Biol Phys. (1992) 23:539–49. doi: 10.1016/0360-3016(92)90009-7 1612954

[98] Nemoto Y Inoue Y Tashiro T Mochizuki K Oda J Kogame S. Intramedullary spinal cord tumors: significance of associated hemorrhage at MR imaging. Radiology. (1992) 182:793–6. doi: 10.1148/radiology.182.3.1535896, PMID: 1535896

[99] De la Fuente C Pumarola M Anor S. Imaging diagnosis—spinal epidural HEMANGIOSARCOMA in a dog. Vet Radiol Ultrasound. (2014) 55:424–7. doi: 10.1111/vru.12074, PMID: 23815770

[100] Mallol C Gutierrez-Quintana R Hammond G Schweizer-Gorgas D De Decker S Novellas R. MRI features of canine hemangiosarcoma affecting the central nervous system. Vet Radiol Ultrasound. (2022) 63:185–96. doi: 10.1111/vru.13041, PMID: 34873768

[101] Barker A Williams JM Chen A Bagley R Jeffery ND. Suspected primary hematomyelia in 3 dogs. Can Vet J. (2015) 56:278–84. PMID: 25750449 PMC4327142

[102] Armstrong JF Perlini M Elbert JA Rissi DR Platt SR. Suspected primary haematomyelia in a French bulldog. J Small Anim Pract. (2021) 62:824. doi: 10.1111/jsap.13343 33908626

[103] Novy J Carruzzo A Maeder P Bogousslavsky J. Spinal cord ischemia: clinical and imaging patterns, pathogenesis, and outcomes in 27 patients. Arch Neurol. (2006) 63:1113–20. doi: 10.1001/archneur.63.8.1113 16908737

[104] Olby NJ da Costa RC Levine JM Stein VM. Canine spinal cord injury C. Prognostic factors in canine acute intervertebral disc disease. Front Vet Sci. (2020) 7:596059. doi: 10.3389/fvets.2020.596059, PMID: 33324703 PMC7725764

[105] Levine GJ Levine JM Budke CM Kerwin SC Au J Vinayak A. Description and repeatability of a newly developed spinal cord injury scale for dogs. Prev Vet Med. (2009) 89:121–7. doi: 10.1016/j.prevetmed.2009.02.016, PMID: 19303151

[106] Abdelaal MA McGuinness FE Sagar G. Case report: spinal extradural haematoma in haemophilia-a — a diagnosis not to be missed. Clin Radiol. (1994) 49:573–5. doi: 10.1016/S0009-9260(05)82941-1 7955874

[107] Hague DW Joslyn S Bush WW Glass EN Durham AC. Clinical, magnetic resonance imaging, and histopathologic findings in 6 dogs with surgically resected extraparenchymal spinal cord hematomas. J Vet Intern Med. (2015) 29:225–30. doi: 10.1111/jvim.12481, PMID: 25619517 PMC4858063

[108] Schouman-Claeys E Frija G Cuenod CA Begon D Paraire F Martin V. MR imaging of acute spinal cord injury: results of an experimental study in dogs. AJNR Am J Neuroradiol. (1990) 11:959–65. PMID: 2121001 PMC8334078

[109] Moriarty HK OC R Moriarty PD Stanley E Lawler LP Kavanagh EC. MR imaging of spinal haematoma: a pictorial review. Br J Radiol. (2019) 92:20180532. doi: 10.1259/bjr.20180532 30407845 PMC6541191

[110] Tidwell AS Specht A Blaeser L Kent M. Magnetic resonance imaging features of extradural hematomas associated with intervertebral disc herniation in a dog. Vet Radiol Ultrasound. (2002) 43:319–24. doi: 10.1111/j.1740-8261.2002.tb01011.x, PMID: 12174993

[111] Hammond LJ Hecht S. Susceptibility artifacts on T2*-weighted magnetic resonance imaging of the canine and feline spine. Vet Radiol Ultrasound. (2015) 56:398–406. doi: 10.1111/vru.12245 25693447

[112] Pierce JL Donahue JH Nacey NC Quirk CR Perry MT Faulconer N. Spinal hematomas: what a radiologist needs to know. Radiographics. (2018) 38:1516–35. doi: 10.1148/rg.2018180099, PMID: 30207937

[113] Hoggard N Wilkinson ID Paley MN Griffiths PD. Imaging of haemorrhagic stroke. Clin Radiol. (2002) 57:957–68. doi: 10.1053/crad.2002.0954 12409105

[114] Ariete V Barnert N Gomez M Mieres M Perez B Gutierrez JC. Morphometrical study of the lumbar segment of the internal vertebral venous plexus in dogs: a contrast CT-based study. Animals (Basel). (2021) 11:1061502. doi: 10.3390/ani11061502 PMC8224572 34067340

[115] Santifort K Beukers M Gil VA Pijnenburg J Van Soens I Mandigers P. Fast three-dimensional contrast-enhanced magnetic resonance angiography of the canine lumbar spinal cord vascular supply: a feasibility study. Vet Radiol Ultrasound. (2022) 63:749–52. doi: 10.1111/vru.13103, PMID: 35569126

[116] Saadoun S Jeffery ND. Acute traumatic spinal cord injury in humans, dogs, and other mammals: the under-appreciated role of the dura. Front Neurol. (2021) 12:629445. doi: 10.3389/fneur.2021.629445, PMID: 33613434 PMC7887286

[117] Park EH White GA Tieber LM. Mechanisms of injury and emergency care of acute spinal cord injury in dogs and cats. J Vet Emerg Crit Care (San Antonio). (2012) 22:160–78. doi: 10.1111/j.1476-4431.2012.00723.x, PMID: 23016808

[118] Zhu F Yao S Ren Z Telemacque D Qu Y Chen K. Early durotomy with duroplasty for severe adult spinal cord injury without radiographic abnormality: a novel concept and method of surgical decompression. Eur Spine J. (2019) 28:2275–82. doi: 10.1007/s00586-019-06091-1, PMID: 31440894

[119] Telemacque D Zhu FZ Ren ZW Chen KF Drepaul D Yao S. Effects of durotomy versus myelotomy in the repair of spinal cord injury. Neural Regen Res. (2020) 15:1814–20. doi: 10.4103/1673-5374.280304 32246622 PMC7513969

[120] Clark R Ferreira A Behr S. Significance of intramedullary T2(*) signal voids in the magnetic resonance imaging of paraplegic deep pain-negative dogs following intervertebral disc extrusion at short-term follow-up. Front Vet Sci. (2023) 10:1248024. doi: 10.3389/fvets.2023.1248024, PMID: 37781293 PMC10533920

[121] Kreppel D Antoniadis G Seeling W. Spinal hematoma: a literature survey with meta-analysis of 613 patients. Neurosurg Rev. (2003) 26:1–49. doi: 10.1007/s10143-002-0224-y, PMID: 12520314

[122] Jakel L Van Nostrand WE Nicoll JAR Werring DJ Verbeek MM. Animal models of cerebral amyloid angiopathy. Clin Sci (Lond). (2017) 131:2469–88. doi: 10.1042/CS20170033 28963121

[123] Van Caenegem N Troupel T Mortier J Thibaud JL Blot S. Suspected spontaneous early hemorrhagic transformation of multiple ischemic strokes secondary to primary splenic torsion in a German shepherd dog. J Vet Intern Med. (2022) 36:2191–8. doi: 10.1111/jvim.16527, PMID: 36106553 PMC9708388

[124] McBride R Rylander H Lyman D. Fibrocartilaginous embolic encephalopathy of the cerebellum and brainstem in a cat. Open Vet J. (2018) 8:489–92. doi: 10.4314/ovj.v8i4.22, PMID: 30775290 PMC6356102

[125] Uchida K Miyauchi Y Nakayama H Goto N. Amyloid angiopathy with cerebral hemorrhage and senile plaque in aged dogs. Nihon Juigaku Zasshi. (1990) 52:605–11. doi: 10.1292/jvms1939.52.605, PMID: 2385040

[126] Munana KR. Encephalitis and meningitis. Vet Clin North Am Small Anim Pract. (1996) 26:857–74. doi: 10.1016/S0195-5616(96)50109-9, PMID: 8813754

[127] Nessler J Wohlsein P Junginger J Hansmann F Erath J Sobbeler F. Meningoencephalomyelitis of unknown origin in cats: a case series describing clinical and pathological findings. Front Vet Sci. (2020) 7:291. doi: 10.3389/fvets.2020.00291, PMID: 32671104 PMC7326087

[128] Flegel T. Breed-specific magnetic resonance imaging characteristics of necrotizing encephalitis in dogs. Front Vet Sci. (2017) 4:203. doi: 10.3389/fvets.2017.00203, PMID: 29255715 PMC5723069

[129] Levine JM Fosgate GT Porter B Schatzberg SJ Greer K. Epidemiology of necrotizing meningoencephalitis in pug dogs. J Vet Intern Med. (2008) 22:961–8. doi: 10.1111/j.1939-1676.2008.0137.x, PMID: 18647157 PMC7166975

[130] DeClementi C Sobczak BR. Common rodenticide Toxicoses in small animals. Vet Clin North Am Small Anim Pract. (2018) 48:1027–38. doi: 10.1016/j.cvsm.2018.06.006, PMID: 30173927

[131] Wood MW Wakim KG Sayre GP Millikan CH Whisnant JP. Relationship between anticoagulants and hemorrhagic cerebral infarction in experimental animals. AMA Arch Neurol Psychiatry. (1958) 79:390–6. doi: 10.1001/archneurpsyc.1958.02340040034003, PMID: 13519943

[132] Losos GJ Crockett E. Toxicity of beril in the dog. Vet Rec. (1969) 85:196. doi: 10.1136/vr.85.7.196, PMID: 5816642

[133] Berry PH Mac Donald JS Alberts AW Molon-Noblot S Chen JS Lo CY. Brain and optic system pathology in hypocholesterolemic dogs treated with a competitive inhibitor of 3-hydroxy-3-methylglutaryl coenzyme a reductase. Am J Pathol. (1988) 132:427–43. PMID: 3414776 PMC1880766

[134] Zook BC. The pathologic anatomy of Lead poisoning in dogs. Vet Pathol. (1972) 9:310–27. doi: 10.1177/030098587200900503 29884005

[135] Luttrell CN Finberg L Drawdy LP. Hemorrhagic encephalopathy induced by hypernatremia. II. Experimental observations on hyperosmolarity in cats. Arch Neurol. (1959) 1:153–60. doi: 10.1001/archneur.1959.03840020027005 14419060

[136] Fonti N Parisi F Aytas C Degl'Innocenti S Cantile C. Neuropathology of central and peripheral nervous system lymphoma in dogs and cats: a study of 92 cases and review of the literature. Animals (Basel). (2023) 13:862. doi: 10.3390/ani13050862 36899719 PMC10000237

[137] Waters DJ Hayden DW Walter PA. Intracranial lesions in dogs with hemangiosarcoma. J Vet Intern Med. (1989) 3:222–30. doi: 10.1111/j.1939-1676.1989.tb00861.x, PMID: 2585369

[138] Jose-Lopez R Gutierrez-Quintana R de la Fuente C Manzanilla EG Sunol A Pi Castro D. Clinical features, diagnosis, and survival analysis of dogs with glioma. J Vet Intern Med. (2021) 35:1902–17. doi: 10.1111/jvim.16199, PMID: 34117807 PMC8295679

[139] Mattei C Oevermann A Schweizer D Guevar J Maddox TW Fleming KL. MRI ischemic and hemorrhagic lesions in arterial and venous territories characterize central nervous system intravascular lymphoma in dogs. Vet Radiol Ultrasound. (2023) 64:294–305. doi: 10.1111/vru.13165, PMID: 36329600

[140] Lipsitz D Higgins RJ Kortz GD Dickinson PJ Bollen AW Naydan DK. Glioblastoma multiforme: clinical findings, magnetic resonance imaging, and pathology in five dogs. Vet Pathol. (2003) 40:659–69. doi: 10.1354/vp.40-6-659, PMID: 14608019

[141] Young BD Levine JM Porter BF Chen-Allen AV Rossmeisl JH Platt SR. Magnetic resonance imaging features of intracranial astrocytomas and oligodendrogliomas in dogs. Vet Radiol Ultrasound. (2011) 52:132–41. doi: 10.1111/j.1740-8261.2010.01758.x, PMID: 21388463

[142] Markovich JE Heinze CR Freeman LM. Thiamine deficiency in dogs and cats. J Am Vet Med Assoc. (2013) 243:649–56. doi: 10.2460/javma.243.5.649, PMID: 23971844

[143] Brenner O Wakshlag JJ Summers BA de Lahunta A. Alaskan husky encephalopathy - a canine neurodegenerative disorder resembling subacute necrotizing encephalomyelopathy (Leigh syndrome). Acta Neuropathol. (2000) 100:50–62. doi: 10.1007/s004010051192, PMID: 10912920

[144] Vandenberghe H Baiker K Nye G Escauriaza L Roberts E Granger N. Diagnostic features of type II fibrinoid leukodystrophy (Alexander disease) in a juvenile beagle dog. J Vet Intern Med. (2023) 37:670–5. doi: 10.1111/jvim.16655, PMID: 36799664 PMC10061190

[145] Willesen JL Langhorn R Nielsen LN. Hemostatic dysfunction in dogs naturally infected with Angiostrongylus vasorum-a narrative review. Pathogens. (2022) 11:249. doi: 10.3390/pathogens11020249 35215192 PMC8878016

[146] Romanucci M Salda LD. Pathophysiology and pathological findings of heatstroke in dogs. Vet Med (Auckl). (2013) 4:1–9. doi: 10.2147/VMRR.S29978, PMID: 32670838 PMC7337213

[147] Bruchim Y Klement E Saragusty J Finkeilstein E Kass P Aroch I. Heat stroke in dogs: a retrospective study of 54 cases (1999-2004) and analysis of risk factors for death. J Vet Intern Med. (2006) 20:38–46. doi: 10.1111/j.1939-1676.2006.tb02821.x, PMID: 16496921

[148] Bruchim Y Loeb E Saragusty J Aroch I. Pathological findings in dogs with fatal heatstroke. J Comp Pathol. (2009) 140:97–104. doi: 10.1016/j.jcpa.2008.07.011 19111315

[149] Church ME Turek BJ Durham AC. Neuropathology of spontaneous hypertensive encephalopathy in cats. Vet Pathol. (2019) 56:778–82. doi: 10.1177/0300985819849500, PMID: 31113291

[150] Whitley NT Corzo-Menendez N Carmichael NG McGarry JW. Cerebral and conjunctival haemorrhages associated with von Willebrand factor deficiency and canine angiostrongylosis. J Small Anim Pract. (2005) 46:75–8. doi: 10.1111/j.1748-5827.2005.tb00296.x, PMID: 15736813

